# Signals of positive selection in genomes of palearctic *Myotis*-bats coexisting with a fungal pathogen

**DOI:** 10.1186/s12864-024-10722-3

**Published:** 2024-09-03

**Authors:** V. G. Twort, V. N. Laine, K. A. Field, F. Whiting-Fawcett, F. Ito, M. Reiman, T. Bartonicka, M. Fritze, V. A. Ilyukha, V. V. Belkin, E. A. Khizhkin, D. M. Reeder, D. Fukui, T. L. Jiang, T. M. Lilley

**Affiliations:** 1 Finnish Museum of Natural History, BatLab Finland, University of Helsinki, Helsinki, Finland; 2 Department of Biology, Bucknell University, Lewisburg, PA USA; 3 Institute of Infection, Veterinary and Ecological Sciences, University of Liverpool, Liverpool, UK; 4 Dept. Botany and Zoology, Faculty of Science, Masaryk University, Kotlarska 2, Brno, 611 37 Czech Republic; 5 Zoological Institute and Museum, University of Greifswald, Greifswald, Germany; 6 German Bat Observatory, Berlin, Germany; 7 Competence Center for Bat Conservation Saxony Anhalt, in the South Harz Karst Landscape Biosphere Reserve, Südharz, Germany; 8 Papanin Institute for Biology of Inland Waters, Russian Academy of Sciences, Borok, Russia; 9 Institute of Biology, Karelian Research Centre, Russian Academy of Sciences, Petrozavodsk, Russia; 10 Graduate School of Agricultural and Life Sciences, The University of Tokyo Fuji Iyashinomori Woodland Study Center, The University of Tokyo, Yamanakako, Japan; 11 Jilin Provincial Key Laboratory of Animal Resource Conservation and Utilization, Northeast Normal University, Changchun, China

**Keywords:** Positive selection, Disease, Fungal pathogen, Bats, Adaptation, Tolerance

## Abstract

Disease can act as a driving force in shaping genetic makeup across populations, even species, if the impacts influence a particularly sensitive part of their life cycles. White-nose disease is caused by a fungal pathogen infecting bats during hibernation. The mycosis has caused massive population declines of susceptible species in North America, particularly in the genus *Myotis*. However, *Myotis* bats appear to tolerate infection in Eurasia, where the fungal pathogen has co-evolved with its bat hosts for an extended period of time. Therefore, with susceptible and tolerant populations, the fungal disease provides a unique opportunity to tease apart factors contributing to tolerance at a genomic level to and gain an understanding of the evolution of non-harmful in host-parasite interactions. To investigate if the fungal disease has caused adaptation on a genomic level in Eurasian bat species, we adopted both whole-genome sequencing approaches and a literature search to compile a set of 300 genes from which to investigate signals of positive selection in genomes of 11 Eurasian bats at the codon-level. Our results indicate significant positive selection in 38 genes, many of which have a marked role in responses to infection. Our findings suggest that white-nose syndrome may have applied a significant selective pressure on Eurasian *Myotis*-bats in the past, which can contribute their survival in co-existence with the pathogen. Our findings provide an insight on the selective pressure pathogens afflict on their hosts using methodology that can be adapted to other host-pathogen study systems.

**Supplementary Information:**

The online version contains supplementary material available at 10.1186/s12864-024-10722-3.

## Introduction

Infectious diseases are natural processes affecting wildlife and contribute to ecosystem stability [1]. However, over the past decades, these processes have been intensified due to anthropogenic activities, such as urbanisation, and environmental and climate change [1–3]. Anthropogenic impacts on the incidence of epizootic diseases have been identified as an increasing threat to wildlife conservation [1, 3]. The role of infectious diseases in population declines and/or extinction has recently received growing recognition, but there is still a critical need to identify and understand the impacts and projections of diseases on populations [4].

White-nose disease, or white-nose syndrome as it is often referred to, (WND/WNS, please see Whiting-Fawcett et al. 2024 [5] for details on nomenclature) is an epizootic caused by a fungal infection affecting hibernating bats in Eurasia and North America [6]. In North America, it is considered one of the most detrimental wildlife diseases of recent decades [7], with mass mortality of affected species causing unprecedented population collapses in many of the affected areas [6, 8] since it was first discovered in the winter of 2006–2007 [6]. The causative agent, the cold-loving fungus *Pseudogymnoascus destructans* (= *Geomyces destructans)* [6], is endemic to the Palearctic, where it does not cause significant mortality in bat hosts [9, 10] due to a potentially extended coevolution between the pathogen and the hosts [11].

Host mortality caused occurs via the disruption of normal patterns of hibernation [12, 13]. Hibernation, characterised by bouts of torpor and arousals, allows bats to survive periods of food scarcity by reducing their energy expenditure via decreased metabolism. Infected North American *Myotis lucifugus* arouse three times more frequently from torpor in the final third of the hibernation period [13], expending large amounts of the fat reserves that are expected to last until insect food is available again in the spring. The psychrophilic fungus invades host tissue during the extended bouts of torpor when the host immune system is downregulated [14]. *Pseudogymnoascus destructans* infection induces the production of inflammatory cytokines during the arousals that take place during hibernation [15–17]. The presumed irritation, such as pain and itchiness, associated with inflammation and immunopathology, in addition to compounds produced by the fungus [18] and evaporative water loss from open sores [19] have been suggested to increase the frequency of arousals. This leads to premature consumption of fat reserves essential for hibernation and death in the more susceptible bat species [12, 13].

In the Palearctic, *P. destructans* infects a number of different bat species, mostly in the genus *Myotis* [9, 20–22]. The coevolution between the fungus and the bat hosts is potentially extensive [11, 23], and although no direct evidence exists, it has been suggested that *Myotis* species may have experienced similar population declines as a result of disease in the past [24]. The most extensively studied Palearctic species, *Myotis myotis*, elicits neither an immediate immune response or more frequent arousals to the infection which prevents the bats from emaciation [25–27], suggesting that these bats utilise tolerance as a response to infection [28, 29]. Tolerance allows the host to evade harmful immunopathology which appears to be a major factor contributing to mortality of bats in the Nearctic [30].

Diseases are a strong driving force of natural selection [31]. Selection pressures result in the modification of the hosts’ genetic diversity and leave behind distinctive signatures in the genome. The nature of these signatures depends on the evolutionary timescale of interest. Among these identifiable footprints of selection are selective sweeps, whereby an advantageous mutation eliminates or reduces variation in the population at linked neutral sites as the mutation increases in frequency. Additionally, alterations in host genetic makeup can be detected in protein coding regions as signals of natural selection; the ratio amino acid changing (non-synonymous or d_N_) to amino acid preserving (synonymous or d_S_) substitutions, also known as the Omega (ω) ratio, is used to infer selection. Under neutral evolution the rates are equal (ω = 1). Whereas, an excess of non-synonymous changes is a sign of positive selection (ω > 1), the converse is associated with a predominant negative selection pressure (ω < 1) [32]. Based on the evidence of a long term association between Palearctic bats and *P. destructans* [23], it is likely that bats have evolved inheritable mechanisms leading to infection tolerance [29]. Signatures of such mechanisms could be detected within protein coding genes associated with wound healing, immune responses and hibernation physiology [15, 26, 27, 33].

Because infected Palearctic *Myotis* bats, overlapping in range with the pathogen, do not experience significant mortality [9, 10] or elicit a transcriptional response to infection [27], we hypothesise that the genomes of these bat species show signs of positive selection in genes that are associated with immune system signalling and function in response to fungal antigens. To investigate the potential impact of extended coexistence with *P. destructans* on the genetic makeup of Palearctic *Myotis* hosts, we use a variety of approaches to detect selection. First, we constructed a whole-genome dataset of 12 *Myotis* species (1 Nearctic, 11 Palearctic) to investigate the general overall patterns of selection in Palearctic *Myotis* and conduct a branch test to detect any lineage-specific selection in either the Palearctic *M. myotis*, which is considered the tolerant main host of *P. destructans* [9], and the susceptible Nearctic *M. lucifugus* sampled prior to the arrival of the pathogen. Second, we also assessed the potential of more recent positive selection with selective sweep analysis using single nucleotide polymorphisms (SNPs) in in the same two species, the Nearctic *M. lucifugus* and Palearctic *M. myotis*. Additionally, we conducted a systematic literature search to list genes already found to be associated with host responses during infection with *P. destructans*. Finally, the combined gene set curated from a literature search, sweep analysis and the phylogenetic branch tests were used to test for the presence of inheritable variation within *Myotis*. The identification of positive selection among these genes may highlight variations that contribute to tolerance among Palearctic lineages of *Myotis*. The identified gene variants and mechanisms contributing to survival in the Palearctic can be applied to conservation genetic approaches to predict the fate of affected populations.

## Methods

### Sample collection, DNA extraction and sequencing

Samples for DNA extraction were obtained from existing collections of the authors. No bats were sampled as a part of the present study.

**Fig. 1:**
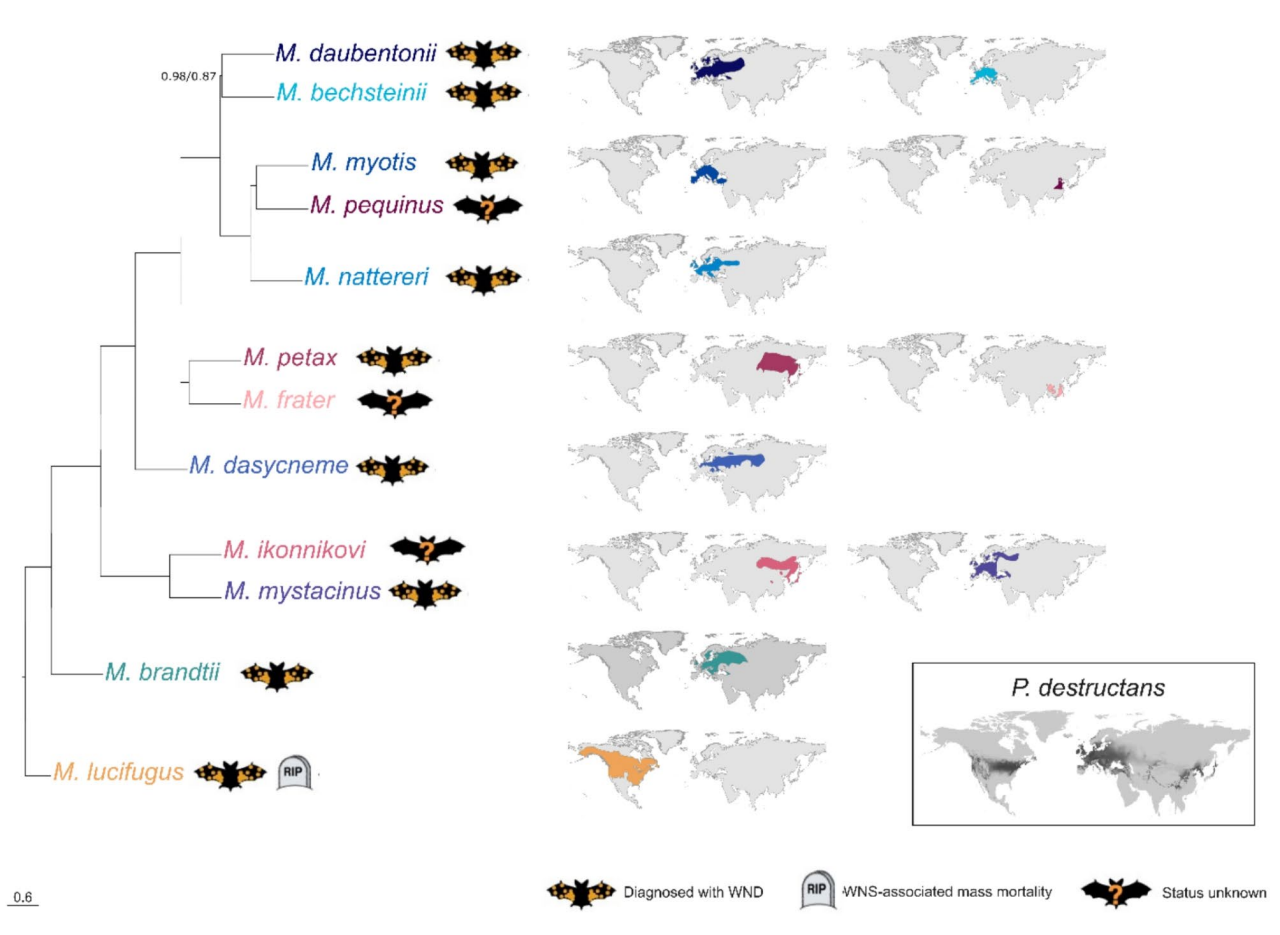
Phylogenetic relationships among 12 *Myotis* species based on species tree reconstruction using 2,515 genes. Support values represent Astral Support/bootstrap values from the two runs of ASTRAL-III, unless otherwise shown all nodes had 1.00/1.00 support. Distribution maps for each species are colour coded according to the taxa on the phylogeny, with the *P. destructans* distribution being shown in grey. The distribution maps are based on IUCN (International Union for Conservation of Nature, https://www.iucnredlist.org Accessed on 03 October 2023). Map of distribution range of *P. destructans* based on Blomberg et al. (2023) [35]. Figure adapted from Harazim et al. 2018 [33]

Eleven out of the twelve species sampled represent the main lineages of *Myotis* from the Palearctic clade (*M. bechsteinii*, *M. daubentonii*, *M. brandtii*, *M. mystacinus*, *M. dasycneme*, *M. frater*, *M. pequinus*, *M. petax*, *M. myotis*, *M. nattereri*, *M. ikonnikovi*) with distributions ranging from the Iberian Peninsula to Japan (See Fig. 1). All species reside in the temperate or boreal zone and utilise extended periods of torpor, hibernation, over the winter months [34]. These species overlap in range with the predicted range of *P. destructans* [35], and all but *M. ikonnakovii* and *M. frater* have been described with the fungal infection, but with no signs of mortality [21]. As a whole, these Palearctic *Myotis* represent taxa that potentially have an extended infection history with the pathogen and are considered tolerant. Of the Palearctic species, the responses to infection by *P. destructans* have been thoroughly documented *M. myotis*, a species considered as the primary host for the fungus [9, 26, 27, 36]. The final species is *M. lucifugus*, the only representative of the Nearctic clade of *Myotis* in our study. The species has been extensively studied with regards to WND [12, 13, 37, 38] and its responses to infection have been documented [15–17, 27, 39–42]. The purpose of this species in our dataset is to act as an outlier against which the Palearctic species are compared. The estimated divergence times for the species within our dataset range from between 19 and 29.5 mya for the split between Nearctic (*M. lucifugus*) and Palearctic (all other species) *Myotis* and c. 5–22 mya within the Palearctic *Myotis* [43]. Full details for each sample are given in Supplementary Table S1.

DNA extraction was carried out using one of the following methods: (1) QIAmp DNA Mini Kit (Qiagen, Germany); (2) DNeasy Blood and Tissue Kit (Qiagen, Germany); (3) SDS Extraction method (full details can be found in Supplementary Methods 1), with extractions being stored at -80 ºC. Library preparation and sequencing was carried out by a variety of providers (see Supplementary Table S1) as follows. For samples processed at: (1) Novogene, United Kingdom: Library preparation was carried out with the Novogene NGS DNA Library Preparation Set (Cat No. PT004) prior to sequencing on a NovaSeq6000 platform to generate 2 × 150 bp reads. (2) Novogene, China: 1.5 µg of DNA was used as input for the TruSeq Nano DNA HT Sample preparation kit (Illumina), with a 350 bp insert. Samples were sequenced on a NovaSeq6000 to generate 2 × 150 bp reads. (3) DNA Sequencing and Genomics Laboratory (BIDGEN): Libraries were prepared using the Nextera™ DNA Flex Library Preparation Kit (Illumina) and sequenced on a NovaSeq6000 to generate 2 × 150 bp reads. (4) University of Liverpool Centre for Genomics Research (CGR): Libraries were prepared using the NEBNext Ultra II FS Kit (~ 300 bp inserts) on the Mosquito platform using a 1/10 reduced volume protocol. Samples were sequenced on a NovaSeq6000 to generate 2 × 150 bp reads. For those samples sequenced at CGR, the provider also carried out read trimming as follows: Adapter removal with Cutadapt (version 1.2.1, [44], followed by trimming with Sickle (version 1.200 [45]), with a minimum window quality score of 20, and removal of reads less than 15 bp.

All raw sequencing reads have been submitted to the NCBI SRA under bioproject PRJNA1051501. In addition to the 18 samples sequenced here, sequencing data from a further six *M. lucifugus*, originally sampled among the first bats in North America to die of the mycosis, at the onset of the white nose-epizootic in North America [46], were downloaded from the NCBI SRA (see Supplementary Table S2).

### Data processing and read mapping

**Fig. 2:**
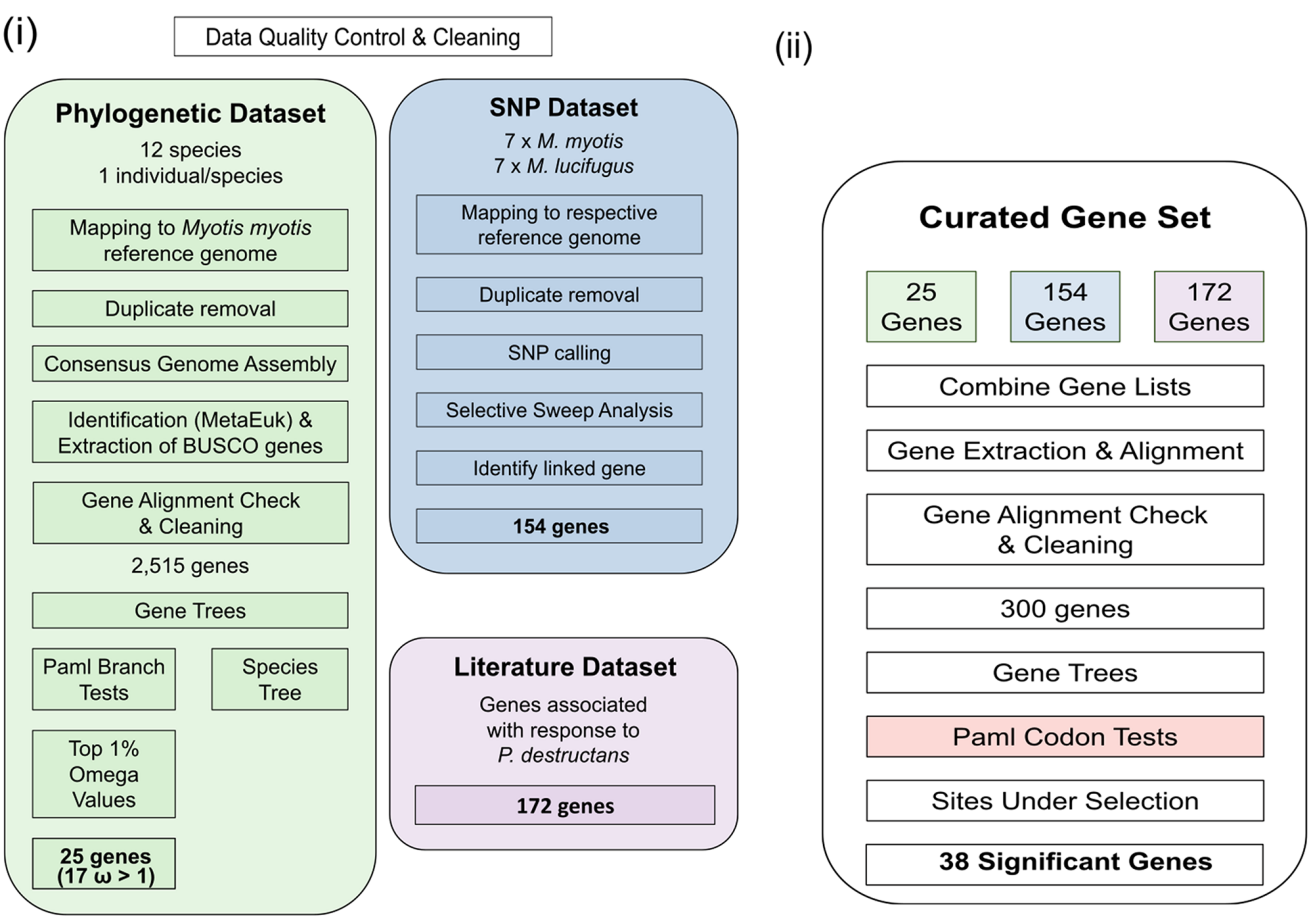
Overall workflow of the three main datasets and their associated analyses. Colour schemes match for Figs. 2 and 3

The study is built on four datasets curated in parallel; (i) phylogenetic dataset; (ii) SNP dataset; (iii) literature search dataset and (iv) curated gene set. An overview of how the four datasets are related and which analyses they were used for is summarised in Fig. 2.

Raw reads were quality checked with FASTQC (version 0.11.8, [47], followed by ambiguous base removal with Prinseq (version 0.20.4, [48]. Adapter removal and quality filtering was undertaken with Trimmomatic (version 0.39, [49], using library appropriate adapter sequences and the following settings: ILLUMINACLIP:< adapter>:2:30:10 LEADING:3 TRAILING:3 SLIDINGWINDOW:4:25 MINLEN:50.

All samples were mapped against the *M. myotis* genome (GCF_014108235.1 mMyoMyo1.p) using bowtie2 (version 2.4.1 [50]) utilising the sensitive-local option. The resulting sam file was converted to a sorted bam file with samtools (version 1.10 [51]). Duplicate removal was carried out with Picard (version 2.27.5 [52]) MarkDuplicates option, with a lenient validation stringency, coordinate sort order and the removal of duplicates. Appropriate reads groups were added to each sample using the AddorReplaceReadGroups function of Picard, with the resulting bam file was indexed with samtools. A consensus assembly was created for each sample with ANGSD (version 0.939, [53] using the following settings: -minQ 20 -doCounts 1 -minMapQ 20 -setMinDepth 3 -dofasta 2.

### Phylogenetic dataset construction and analysis

#### Orthologue identification

In order to identify single copy orthologues, the BUSCO Mammalia_odb10 reference dataset [54] was used. At the time of analysis, the current version of BUSCO did not output nucleotide sequences, therefore Metaeuk (Release 5-34c21f2, [55]) was used to identify gene sequences (settings: easy-predict –cov 0.6 –filter-mas 1 –metaeuk-eval 0.0001 -metaeuk-tcov 0.6 –min-length 40). Result files were filtered to remove any duplicated genes and genes identified in less than 8 individuals. Both the protein and nucleotide sequences produced by Metaeuk were taken forward.

Initial protein alignments were generated using MAFFT (version 7.429, [56]), using the ‘auto’ option and manually checked to ensure accuracy, screened for the presence of pseudogenes, reading frame errors and alignment errors using Geneious 11.0.3 (https://www.geneious.com). After screening, any alignments with less than 8 sequences, < 50% of the ORF or those lacking either *M. myotis* or *M. lucifugus* sequences were discarded. This resulted in a final dataset of 2,515 genes (Supplementary Table S3). Amino acid alignments were converted into codon alignments utilising the nucleotide sequences extracted by Metaeuk with Pal2Nal (Version 14, [57]). Alignments were cleaned with gblocks (0.91b, [58]) with the following settings: -t = c, -b3 = 8, -b5 = 15 -b5 = a, b1 = 50% of the number of sequences + 2 and b2 = 50% of the number of sequences + 4. The final gene alignments can be accessed at Zenodo DOI: 10.5281/zenodo.11353518. Annotations for each BUSCO gene code were retrieved from OrthoDB v10 [59].

#### Phylogenetic analysis

To reconstruct the evolutionary relationships a multispecies coalescent analysis was used. Individual gene trees were reconstructed with IQ-TREE2 (2.1.3, [60]), with ModelFinder [61] being run to determine the optimal model and 1000 ultrafast bootstraps (UFBoot2) approximations [62]. For species tree inference two approaches were undertaken with ATSTRAL-III (5.7.8, [63]), first default parameters were used, while the second analysis incorporated the bootstrap trees from IQ-TREE2 with 500 replicates.

#### Inferring selection associated with *M. myotis* and *M. lucifugus*

Phylogenetic branch tests were implemented to detect selection acting on particular lineages. Two independent analyses were carried out, in the first analysis the branch leading to *M. myotis* was labelled as the foreground branch, while in the second the branch leading to *M. lucifugu*s was considered the foreground. For each analysis individual gene trees were used. In order for the labelled branches to be read correctly, all of the extra information associated with each branch, such as the branch length and bootstrap scores, were removed with PareTree (version 1.02, Available from: http://emmahodcroft.com/PareTree.html*)*, followed by the branch of interest being labelled. Branch models allow for the labelled (or foreground) branch to have an ω independent of the rest of the phylogeny and were carried out with Paml 4.9j [64]. P-values were generated from the LRT tests using the R software (version 4.1.2, [65]) function pchisq, with correction for multiple tests being carried out with the holm-bonferroni correction using the p.adjust function.

### SNP dataset construction and analysis

#### SNP calling

In parallel to the phylogenetic dataset and branch tests (see Fig. 2.), SNPs were called from *M. myotis* and *M. lucifugus*, seven individuals from each species, for selective sweep detection. Because of low numbers of individuals and low sequencing coverage in many of the individuals, we used two SNP callers after which overlapping SNPs were extracted. For *M. myotis* we used the aligned deduplicated files with read groups (see Data processing and read mapping) for both callers. We also aligned *M. lucifugus* samples to a draft chromosome-level *M. lucifugus* assembly (mMyoLuc1, NCBI BioProject PRJNA973719) using the same settings as the *M. myotis* samples (see Data processing and read mapping), with deduplication and read group addition. The first caller was ANGSD version 0.940 [53, 66] with settings -uniqueOnly 1 -remove_bads 1 -only_proper_pairs 1 -C 50 -baq 1 -minMapQ 20 -minQ 20 -minInd 7 -doCounts 1 -setMinDepth 10 -setMaxDepth 100 -GL 2 -doMajorMinor 1 -doMaf 1 -SNP_pval 1e-6 -doPost 2 -doGeno 3 -doBcf 1 -doGlf 2. The second SNP caller was GATK HaplotypeCaller version 4.3.0.0 [53] with -ERC GVCF and by scaffold (-L). The scaffolds were combined with picard’s GatherVcfs function. All the individual vcfs were combined with GATK’s CombineGVCFs tool. The variants were genotyped with GATK GenotypeGVCFs with settings --heterozygosity 0.001 and -stand-call-conf 20 and SNPs selected with GATK SelectVariants with settings --select-type-to-include SNP --restrict-alleles-to BIALLELIC. SNPs were filtered GATK’s VariantFiltration with --filter-expression “QD < 2.0 || FS > 60.0 || MQ < 40.0 || MappingQualityRankSum < -12.5 || SOR > 3.00 || ReadPosRankSum > -8.00” --filter-name “snp_filter” and selected from the files with vcftools --remove-filtered-all, version 0.1.16 [67].

Both SNP caller files from both species were filtered with vcftools using settings --min-alleles 2 --max-alleles 2 --minDP 5 --maxDP 100 --max-missing 1. In order to select the overlapping SNPs from the files, we used Bedtools intersect version 2.30.0 [68] (bedtools intersect -u -a GATK.vcf -b ANGSD.vcf) and filtered the overlapping SNPs with vcftools --positions. Finally, the SNPs with differing genotypes within an individual from the overlap set was first detected with vcftools --diff-site-discordance and removed with vcftools --exclude-positions.

#### Selective sweep detection

For the selective sweep detection we used RAiSD version 2.9 [69] excluding known genome gaps. RAiSD was run for each species and the resulting outlier windows (highest µ values 0.05%) were filtered using a conservative threshold (α = 0.0005) in R version 4.3.0. The genes from these windows that were above the threshold were extracted with bedtools intersect using *M. myotis* NCBI gene annotation release 100 version GCF_014108235.1_mMyoMyo1 in *M. myotis*. In *M. lucifugus* samples a draft annotation was used. The mMyoLuc1 draft assembly was annotated by porting over gene annotations from mMyoMyo1 using Liftoff (v1.6.3, [70] and the settings “-exclude_partial -cds -polish;” these annotations were further cleaned using AGAT (v0.9.2, [71]).

#### Nucleotide diversity

Nucleotide diversity, Watterson Theta, and Tajima’s D were estimated for both species separately using ANGSD. First, the dosaf 1 function was used to calculate the site allele frequency spectrum likelihood (saf) for each species based on individual genotype likelihoods using the same quality specification as in genotype calling. Then, the realSFS function was used to optimize the saf and estimate the unfolded site frequency spectrum (SFS; [72]). Nucleotide diversity, Watterson Theta, and Tajima’s D were calculated for each site with the commands saf2theta and thetaStat in ANGSD.

### Literature search

We carried out a literature search to identify genes previously determined to be linked to White-Nose tolerance, using Web of Science (Clarivate) (carried out on 17/04/2023). The presence of the following keywords was searched for in the abstract or title or keywords: ((gene OR genome OR genetic OR transcript*) AND (*Pseudogymnoascus destructans* OR WNS OR white-nose syndrome OR white-nose disease)). The search yielded 134 papers. Upon closer investigation, 11 of these papers looked at bat genetic changes associated with the fungal disease, yielding a total of 172 genes (Supplementary Table S4 A).

### Curated gene dataset

#### Gene identification, extraction and alignment

A curated gene dataset included the genes identified in the literature search, the genes identified in the sweep analysis (see Supplementary Table S4B) and the genes with the top 1% of ω values (from the phylogenetic dataset, see Sect. 3.3 and Supplementary Table S4 C). Gene codes, and aliases were checked against GeneCards [73]. This resulted in a total of 347 genes of interest (Supplementary Table S4D).

To extract the coding (CDS) region of each gene, the *M. myotis* NCBI gene annotation release 100 version GCF_014108235.1_mMyoMyo1 was searched for corresponding CDS entries. In the case where genes had multiple isoforms, the longest version was extracted. Exon extraction from the mMyoMyo1 genome was carried out using bedtools getfasta (v2.30.0 [68]) with the force strandedness option, followed by concatenation to create a reference CDS sequence. To ensure correct assembly of each gene, a blastp search was carried out against the NCBI nr database. These assembled CDS sequences were used as a reference set, to ensure correct extraction and assembly of sample CDS regions.

Exons were extracted from each consensus assembly using bedtools getfasta using the force strandedness option and assembled against the reference sequence using the ‘assemble to reference’ option in Geneious, with the subsequent CDS assemblies being aligned using MAFFT, using the auto option. Alignments were manually screened for frameshifts and difficult to align regions, those with less than 8 sequences, < 80% of the CDS region and those lacking sequences from *M. lucifugus* and *M. myotis* were discarded. This resulted in a final dataset of 300 genes (Supplementary Table S5). Alignments were automatically cleaned with gblocks with the same settings as the phylogenetic dataset (See orthologue identification). The final gene alignments can be accessed at Zenodo DOI: 10.5281/zenodo.11353518.

#### Curated gene set site selection patterns

To detect patterns of selection within individual genes, site-based likelihood models were used, whereby ω values vary among codons but not branches, therefore the ω for any given codon is averaged across all branches in the phylogeny and does not indicate which species adaptation is occurring in. For each gene, individual gene trees were first constructed under the maximum likelihood framework with IQ-TREE2, with model selection with ModelFinder and 1000 UFBoot2 approximations. For tests of selection, the ω ratio was estimated for each gene using the CODEML package of PAML, with individual gene trees being used. To detect amino acid sites under selection, four-site model comparisons were implemented (M0:M3, M1a: M2a, M7:M8, M8a: M8). These models have been extensively described elsewhere [64]. Briefly, the one ratio model (M0) allows for a single ω for all sites. The nearly neutral (M1a) has two categories of sites, ω = 1 and ω < 1. M2a (positive selection) has the same two categories as the M1a, with the addition of an ω > 1 category. The discrete (M3) model has three categories of sites for which ω can vary. M7 (‘beta’ neutral model) has eight site categories with eight ω taken from a discrete approximation of the beta distribution (range 0–1), meaning that the signal for positive selection cannot be detected. M8 (‘beta’ plus ω) has the same eight categories as the M7 model, plus and additional categories for which ω can vary from 0 to > 1. While the M8a (beta plus ωS = 1) model is similar to the M8 model, with the ω_1_ being fixed at one. Multiple models and comparisons were used to test the robustness of the patterns observed. The M0:M3 tests whether ω varies between sites. The other comparisons test for positive selection, with the M1a: M2a being a more conservative test, the M7:M8 having more power and the M8a: M8 combining both power and robustness. Likelihood ratio tests (LRTs) between nested models, where ω is allowed to vary above one and the associated null model, allows inference of the selection pressures acting along the protein sequence [64]. P-values were generated from the LRT tests using the R function pchisq, with correction for multiple tests being carried out with the holm-bonferroni correction using the p.adjust function. Codons under positive selection were identified using the BEB (Bayes Empirical Bayes) method under the M2a model.

For visualisation of positively selected sites within the ANXA1, TNFSF4, CXCL16 and ANKRD17 protein structures, Phyre2 [74] was used to predict protein models using a reference bat protein sequence (XP_036186722.1, XP_036199699.1, XP_036196999.1, XP_036191841.1). Where reliable models could be constructed, the resulting structure was used to predict relative solvent accessibility (RSA) with PolyView-2D [75]. Binding sites in the ANXA1 protein were identified by comparison against the Rat (*Rattus norvegicus*) version of the protein (Uniprot Id: P07150). For transmembrane domain containing proteins, CXCL16 and TNFSF4, the location of transmembrane domains and signal peptides was carried out with Protter [76].

### Gene ontology

Functional enrichment was tested using g: Profiler v. e109_eg56_p17_773ec798 [77]. Enrichment tests for both overrepresentation and underrepresentation were performed using the list of 2,515 genes (Phylogenetic Dataset). Overrepresentation enrichment was analysed for the 38 genes identified as under selection in the Curated Gene Set. For all comparisons, human annotations were used with the background list of all annotated genes. GO enrichment was measured using a g: SCS multiple testing correction threshold of 0.05 and the GO: BP, KEGG, Reactome, and WikiPathways databases.

## Results

We generated low coverage whole genome data for 18 *Myotis* specimens, encompassing 12 species (see Fig. 1 for distributions of host species and *P. destructans* infections). In addition, a further six *M. lucifugus* samples were downloaded from genbank (Supplementary Table S2). Mapping to the *M. myotis* reference genome resulted in an average mapping rate of 96% (Range: 85 − 99%) and an average genomic depth of coverage of 18X (Range: 6X − 53X). See Supplementary Table S6 for full sequencing statistics.

### Phylogenetic dataset

Screening of single copy orthologues among our 12 *Myotis* species, resulted in a final dataset of 2,515 genes. Gene completeness ranged from 82 to 100%, with 2,173 of these genes being found in all 12 species (Supplementary Table S3). When this list of 2,515 genes was tested for functional enrichment (Supplementary Fig. S1), we found that some significant enrichment was observed, but mostly for broad functional categories, such as GO:0050794 regulation of cellular process (padj = 2.632 × 10^–17^) and GO:0140053 mitochondrial gene expression (padj = 4.751 × 10^–7^). We also tested this gene list for underrepresentation and found that certain categories were significantly underrepresented, such as GO:0000244 spliceosomal tri-snRNP complex assembly (padj = 1.790 × 10^–52^) and GO:0007186 G protein-coupled receptor signalling pathway (padj = 2.978 × 10^–4^), but no immune-related categories were found to be underrepresented (Supplementary Fig. S2). We conclude that this list was not strongly biased in favour of any particular subset of functional pathways and that various immune pathways were sufficiently represented.

To ensure the evolutionary relationships between our samples were as expected the 2,515 gene trees were used to reconstruct the species tree (Fig. 1), which resulted in a topology with generally high support. Astral support values of 100% were estimated for all nodes, except for the split between *M. daubentonii* and *M. bechsteinii*. Generally speaking, reconstructed relationships agree with previous studies [43, 78–80], with the primary difference in our topology being that *M. pequinus* is recovered as the sister species to *M. myotis* rather than *M. nattereri*.

To test for differences in selection in the branches leading to either *M. myotis* or *M. lucifugus*, independent branch tests were carried out. In all tests, no genes were found to have a statistically significant difference between the foreground and background ratios (Supplementary Table S7). A comparison of ω values under the neutral model for the phylogeny as a whole revealed strong patterns of purifying selection (ω < 1), with 17 genes having an ω value > 1 (average: 0.20, Range: 0.0001–2.12, Supplementary Fig. 3A, Supplementary Table S7). The gene with the highest ω value was *TNFSF4* (BUSCO code:188443at40674, ω of 2.12).

### SNP dataset: selective sweeps detection and nucleotide diversity

In addition to the multi species phylogenetic dataset and consecutive branch tests, we also carried out a selective sweep analysis for both *M. myotis* and *M. lucifugus* (See Fig. 2.). We called 1,438,586 SNPs for *M. lucifugus* and 3,915,084 SNPs for *M. myotis* after filtering with ANGSD, and 4,304,169 and 11,757,539 SNPs with GATK, respectively (Supplementary Table S8). Of these, the final set of overlapping SNPs was for *M. lucifugus* 927,923 SNPs, and for *M. myoti*s 3,005,290 SNPs (Supplementary Table 8). In *M. lucifugus*, there were 464 outlier selective sweep windows which covered 17 genes (Supplementary Fig. S4, Supplementary Table S9). In *M. myotis*, there were 1,499 outlier windows and 174 genes (Supplementary Fig. S5, Supplementary Table S10). Three outlier genes were shared between the two species: *ARHGEF4*,* PLSCR1* and *PLOD2*. Nucleotide diversity, Watterson Theta, and Tajima’s D were higher in *M. myotis* than in *M. lucifugus* (Supplementary Fig. S6A). Furthermore, some of the sweep regions matched with the low diversity regions in the whole genome level diversity plots (Supplementary Fig. S6A and B). When unidentified LOC-genes were removed, total of 154 sweep genes were added to the curated gene list (see below).

### Curated gene set: codon test results

**Fig. 3:**
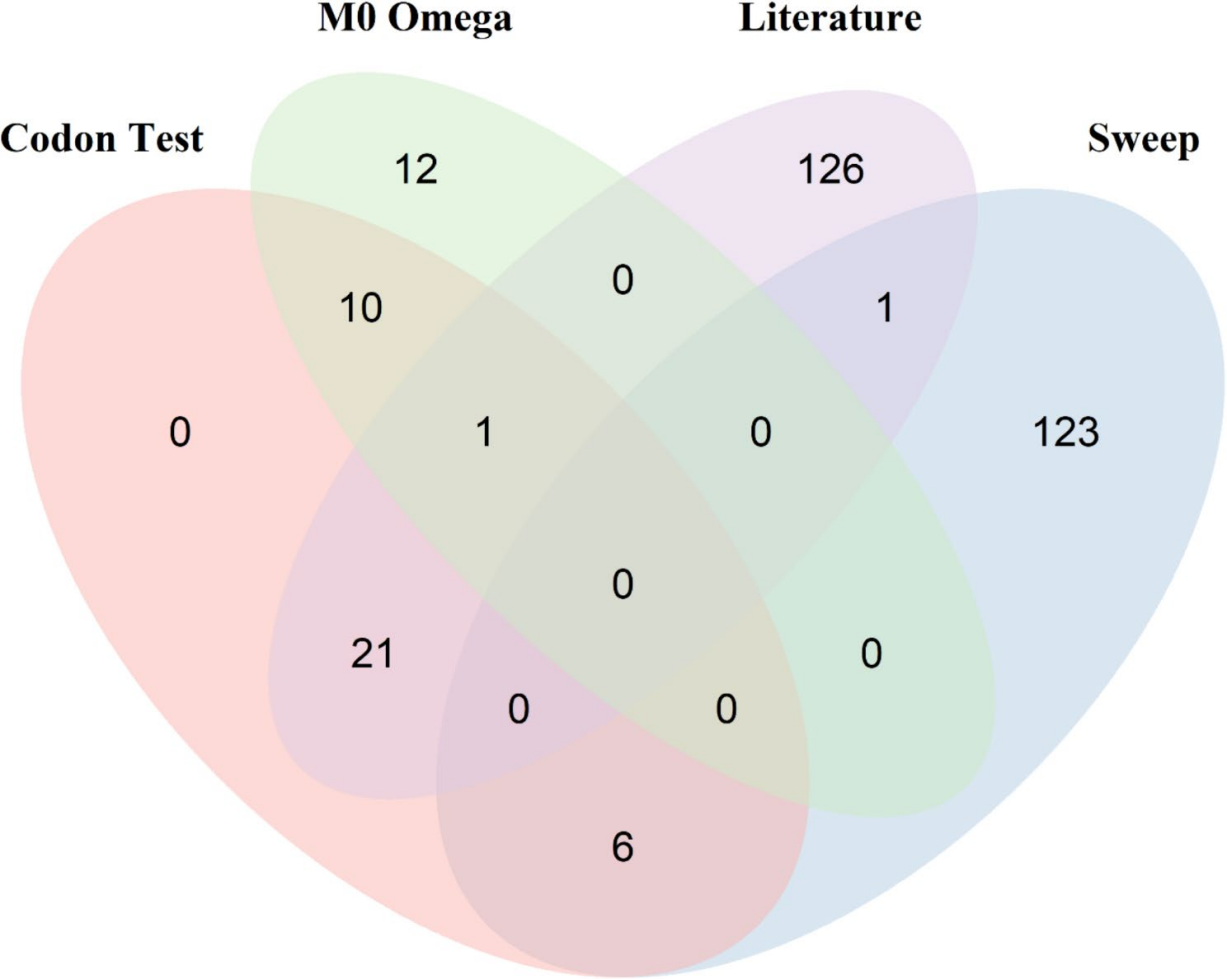
Venn Diagram of the curated gene set (300 genes in total) highlighting which dataset(s) they originate from and which analysis, if any, where they are significant. M0 Omega refers to the top 1% of omega values from the branch test null model (Phylogenetic Dataset); Sweep refers to the genes linked to the SNPs identified in our selective sweep analysis; Literature refers to all the genes identified from our literature search; and Codon Test refers to the 38 genes identified as under positive selection from our curated gene dataset

A final gene set of 300 protein coding genes were identified and extracted from our dataset. These 300 genes consisted of those genes identified in: (I) Literature search, (II) Selective Sweep analysis and (III) overall ω values from the phylogenetic dataset. An overview of how these datasets are related is shown in Fig. 2, and the overlap between each of these categories is shown in Fig. 3.

Of the 300 genes, 270 were identified in all 12 species (Supplementary Table S5). To determine the selective pressures acting on these genes, an ω value was calculated for each orthologous gene set using site-based likelihood models. We found the majority of genes to be evolving under purifying selection when a single ω value is calculated across the entire coding region using the one-ratio (M0) model, mean = 0.32, range 0.0001–2.12 (Supplementary Fig. S3B, Supplementary Table S11). Twenty-one genes have an ω > 1, indicating the protein as a whole is under positive selection (Supplementary Table S12 A). As seen in the phylogenetic dataset, *TNFSF4* had the highest ω of 2.12. Rate heterogeneity among sites was tested using the M0:M3 comparison; significant among site variation was observed in 107 of the 300 genes tested. This, however, is not a test of positive selection. The three different model comparisons allow for the identification of positive selection among codons, each varying in their power and robustness, with ω values for each codon being averaged across the phylogeny. The genes identified in each comparison are given in Supplementary Table S12B. A total of 46 genes were identified in one or more comparisons, with 38 genes being identified in all three tests. In these 38 genes showing the strongest evidence for positive selection, we identified specific amino acids sites under significant selection. The number of sites identified as statistically significant under the robust M2a model ranges from 0 to 46 for each gene (Supplementary Table S13). Of the 38 genes showing the strongest signal for selection, six were from the selective sweep genes, 11 from the top 1% of omega values and 22 from the literature search (Fig. 3).

**Fig. 4:**
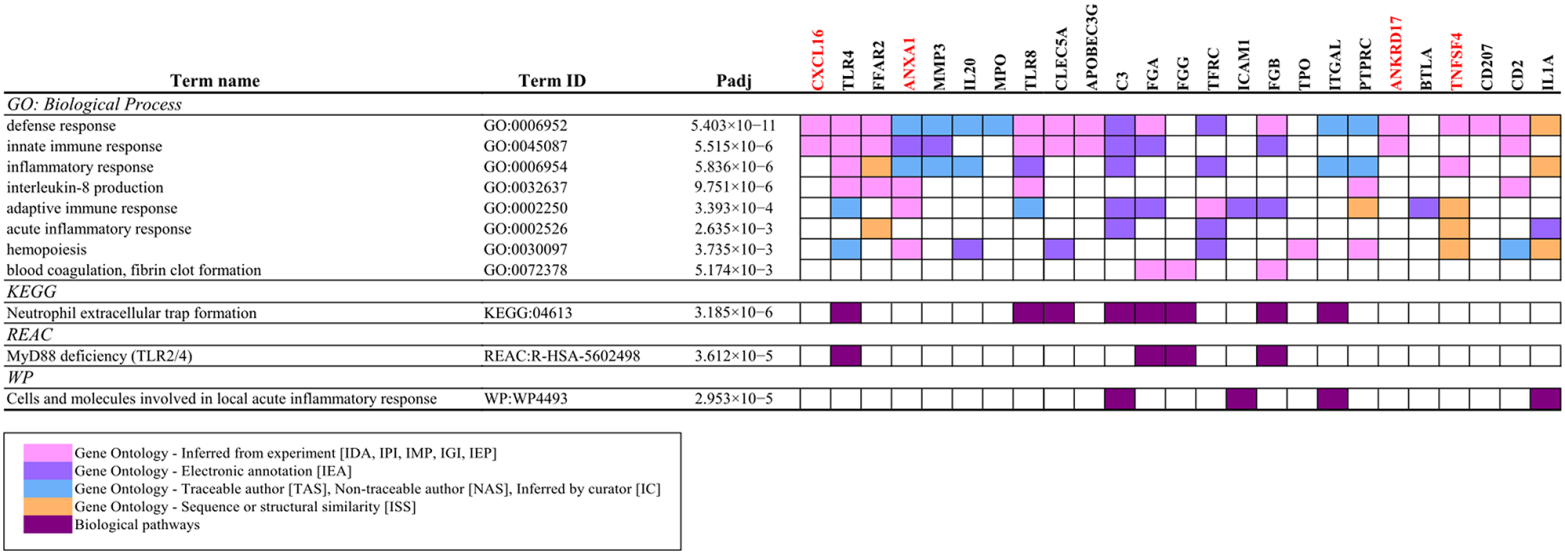
An overview of some of the key categories enriched in our 38 genes under positive selection. The four genes discussed in further detail are highlighted in red

The 38 genes that were identified with the strongest evidence for selection were further examined for functional enrichment using a statistical enrichment analysis and human gene ontology databases. We found that this gene list was highly enriched for genes with functional annotations related to many immune-related categories (Supplementary Table S14) and categories related to inflammatory immune responses were particularly common (Fig. 4). For example, in the GO: Biological Process database, we found enrichment of GO:0045087 innate immune response (padj = 5.515 × 10^− 6^), GO:0006954 inflammatory response (padj = 5.836 × 10^− 6^), GO:0032637 interleukin-8 production (padj = 9.751 × 10^− 6^) GO:0002250 adaptive immune response (padj = 3.393 × 10^− 4^), and GO:0030097 hemopoiesis (padj = 3.735 × 10^− 3^). In the KEGG database, we found enrichment for category KEGG:04613 Neutrophil extracellular trap formation (padj = 3.185 × 10^− 6^); in the Reactome database, we found enrichment for categories involved in the TLR2/4 pathway, such as REAC: R-HSA-5,602,498 MyD88 deficiency (TLR2/4) (padj = 3.612 × 10^− 5^); and in the WikiPathways database, we found enrichment of category WP: WP4493 Cells and molecules involved in local acute inflammatory response (padj = 2.953 × 10^− 5^). Together, these functional enrichment results demonstrate that the majority of the genes identified under the strongest selection by our screens are related to inflammatory immune responses to the fungal pathogen mediated by both innate and adaptive mechanisms.

**Fig. 5:**
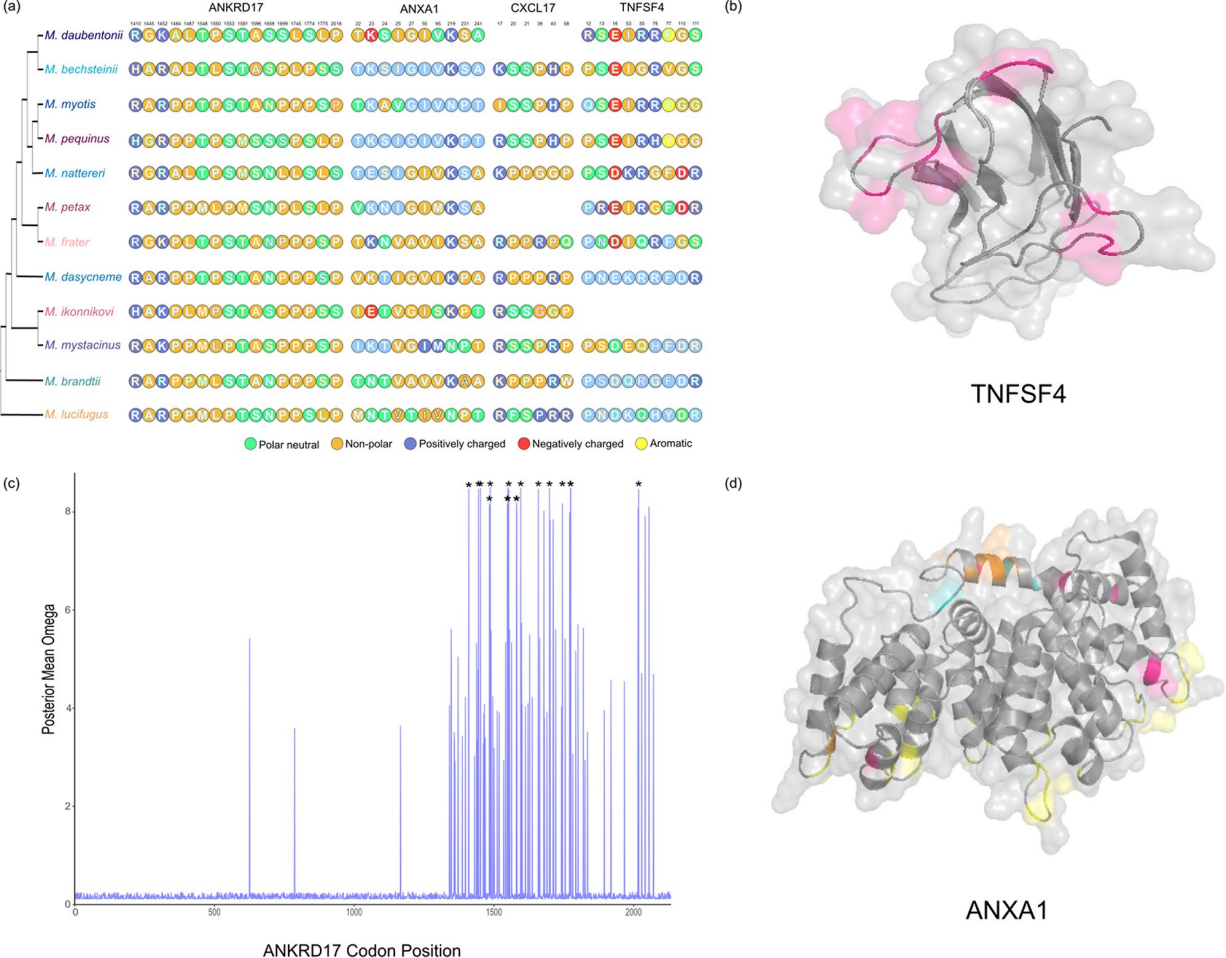
(**A**) An overview of the sites under positive selection and their amino acid changes relative to their phylogenetic position for our four proteins (ANKRD17, ANXA1, CXCL16, TNFSF4) of interest. The phylogenetic tree shown is the same as that shown in Fig. 1. (**B**) Three-dimensional model of TNFSF4, with the residues under selection being highlighted in pink. (**C**) Plot of omega value along the length of the ANKRD17 protein, positions with an * are those identified as being under positive selection. (**D**) Three-dimensional model of ANXA1, with the calcium binding sites being shown in yellow, sites under positive selection have been coloured as follows: Pink: Codons under selection in our study; Orange: Codons under selection in both this study and Harazim et al. 2018 [33]; Aqua: Codons under selection in Harazim et al. 2018 [33]

The gene, *TNFSF4* had the highest overall ω in the Phylogenetic and Curated Gene datasets, with an overall ω of 2.12. In addition, 9 sites were identified in our codon tests as being under positive selection, with ω values of ~ 9 (Supplementary Table S13, Fig. 5A). All nine sites are located in the extracellular portion of the protein (Supplementary Fig. S7). Modelling of the three dimensional protein structure was successful for ~ 60% of the protein sequence (Supplementary Model 1, Fig. S5B, a 360^o^ view is shown in Supplementary File S1); assessing the solvent accessibility of the resulting model showed that residue 121 is fully exposed to the solvent, with an additional 3 sites (22, 64, 66) at least partially exposed, with RSA values > 5, and the remaining 5 being mostly buried within the protein structure (RSA < 5, Supplementary Fig. S8).

Among the 38 genes with strong evidence of positive selection, the gene *CXCL16* was the only gene to have originated from multiple datasets (being identified in the literature screen [27] and among the genes with an ω > 1 in the Phylogenetic dataset). The protein as a whole has an ω of 1.4, with six codons being identified as being under positive selection (ω range: 4.6–4.8, Supplementary Table S13, Fig. 5A). We were unable to model the structure of *CXCL16* with high confidence, however all six sites occur within the extracellular region of the protein (Supplementary Fig. S9).

Of the 11 sweep genes also identified in the codon analysis, *ANKRD17* had the largest proportion of codons under positive selection. Overall, *ANKRD17* had an ω of 0.3. A total of 16 codons were identified as being under positive selection all with an ω value of ~ 8 (Supplementary Table S13, Fig. 5A). All 16 codons occur towards the end of the coding region (Fig. 5C), with very little variation seen in approximately the first half of the coding sequence (Supplementary Fig. S10). It was not possible to model the structure of this protein with high confidence, so no inference on where the 16 codons are in the three-dimensional structure is possible.

Annexin A1 (*ANXA1)* was among the genes with positively selected sites, that had previously been identified in other studies [27, 33], with some evidence for codon specific selection occurring. Overall, *ANXA1* had an ω of 0.5, with 10 codons being identified as under positive selection with ω values of ~ 7.8 (Supplementary Tables 12 and 13, Fig. 5A). Half of the sites identified are located in the N-terminal region of the protein (5 out of 10 sites identified are within the range 22–27). Relative solvent accessibility calculations based on the modelled structure (Supplementary Model 2, Fig. S5D, a 360^o^ view is shown in Supplementary File S2), show residue 27 is fully exposed to the solvent, while residues 22, 23 and 219 are partially exposed (RSA ≥ 5) and the remaining residues are either completely or mostly buried within the protein (Supplementary Fig. S11).

## Discussion

Our comprehensive genomic study, utilising candidate genes sourced from a variety of datasets, detected strong positive selection in a suite of genes in Palearctic species of *Myotis*. Although a myriad of different factors may contribute to selection pressures across the genome in our focal species, a considerable proportion of the genes have been associated with responses to fungal infection.

The species tree generated in this study using 2,515 genes generally agrees with the more recent studies on *Myotis* relationships [43]. One should bear in mind, that the tree produced for this study is limited due to the scope of the study and number of species used, and no novel results on evolutionary relationships can be inferred. Investigation into patterns of selection presents a difficult statistical challenge, which can be influenced by tree topology [81]. We acknowledge that the use of gene trees, as opposed to the species tree, may influence our results. However, we believe the use of gene trees to be appropriate for a number of reasons. Evolutionary relationships among *Myotis* species have been shown to be discordant [82–84]. Secondly, constraining the topology for a given gene can overestimate the substitution rate on constrained branches, which in turn overestimates the ω ratio, leading to an increase in false positives [85] Indeed, within our dataset despite the high branch support for relationships, the conflict between gene trees and species trees is high. The level of conflict can be assessed with quartet scores, which show an overall score of 0.79 for the species tree topology, however some branches have scores as low as 0.34 (Supplementary Fig. S12), emphasising the high level of discordance among *Myotis* species relationships. We believe by not constraining our phylogenetic branch tests to the species tree topology we provide a more conservative estimation of selection patterns.

The patterns of selection on the proteins in the phylogenetic dataset highlight overall functional constraint (ω < 1). This is a common occurrence with proteins generally being under purifying selection as a whole, with adaptation occurring within proteins at selected sites/regions [86, 87]. Of the 2,515 genes tested, 17 had overall ω values indicative of positive selection. Of these genes showing positive selection, the strongest signal was detected in *TNFSF4*. However, we found no evidence for lineage specific selection associated with either *M. myotis*, the species most associated with infection in the Palearctic [9, 21] or *M. lucifugus*, an affected species in the Nearctic [8], when these were contrasted against the other *Myotis* species in a branch test. From this analysis, the top 1% of highest ω values were then included in the subsequent curated gene set.

We also looked at signals of selection using selective sweep analysis in European *M. myotis*, using naïve *M. lucifugus* sampled at the onset of the epizootic in North America as a comparison. Over a hundred genes were outliers in the *M. myotis* population (Fig. 2, Supplementary Fig. S5) that has coevolved with the fungal pathogen, in contrast to the *M. lucifugus*, where only seventeen genes were in the sweep regions (Supplementary Fig. S4). Three genes were shared between the species, most notably phospholipid scramblase 1 (*PLSCR1*), an important gene in the antiviral responses [88]. It is important to note that while selective sweep analyses can provide valuable insights into the genetic basis of adaptation, they have limitations, and the conclusions drawn should be interpreted with caution. In our case, sample size and sequencing coverage were the most important factors limiting the analyses. Other evolutionary processes and demographic factors as well can also influence patterns of genetic diversity, and it can be challenging to distinguish the causality between these. Therefore, multiple lines of evidence and additional studies are often necessary to confirm the effects of selection on specific loci.

We constructed a curated gene set of 300 proteins from the literature search, sweep analysis and overall ω values to investigate selection patterns within genes potentially associated with tolerance to infection by *P. destructans*. As with our phylogenetic dataset, most genes had an ω < 1, indicative of overall purifying selection. Twenty-one genes including *TNFSF4* showed signals for positive selection over the whole gene. Among our 300 genes, we found evidence for selection acting on at least one amino acid site in 38 genes.

We found that *TNFSF4* showed the most positive selection amongst the genes analysed in our study. Interestingly, this gene did not originate from the candidate set of genes gleaned from literature on WND, but rather from the phylogenomic analysis that took an unbiased approach to identifying genes for our downstream analyses. This protein, tumor necrosis factor (ligand) superfamily, member 4, also known as OX40L or CD252, is a potent activator of inflammatory signalling when it is expressed on antigen-presenting cells where it can activate OX40 on T lymphocytes, NK cells, neutrophils, and others [89]. TNFR/TNF superfamily members can control diverse aspects of immune function. The mostly extracellular protein that is encoded by *TNFSF4*, in conjunction with its partner TNFRSF4, is associated with strong regulation of conventional CD4 and CD8 T cells, modulation of NKT cell and NK cell function, and mediation of cross-talk with professional antigen-presenting cells and diverse cell types such as mast cells, smooth muscle cells and endothelial cells. Additionally, TNFSF4-TNFSRF4 interactions alter the differentiation and activity of regulatory T cells [89] and TNFSF4 mediates adhesion of activated T cells to endothelial cells during infection, inducing secretion of proinflammatory cytokines [90]. This gene clearly has a diverse function in the immune system, including that of viral immunity, but has also been specifically linked to responses to fungal infections. Several investigations have demonstrated that *TNFSF4* expression is upregulated in mice sensitized to *Aspergillus fumigatus* [91] and that *TNFSF4* polymorphisms are associated with the risk of developing invasive aspergillosis infection [92]. In addition, blocking TNFSF4, either transcription or translation, has produced strong therapeutic effects in multiple animal models of autoimmune and inflammatory disease [89]. Because of the diverse function of the TNFR/TNF superfamily, we cannot associate positive selection in the gene to just extended exposure to *P. destructans*, as it may also be associated with coevolution with viruses [93].

*CXCL16* is another notable gene, filtering through to the final dataset from both the top 1% of genes in the phylogenetic dataset and the literature search, being previously observed as an upregulated gene in naïve infected *M. lucifugus* [27]. The protein, expressed as either a soluble or a transmembrane form, is a marker of inflammation in humans and is produced by monocytes/macrophages, B cells, dendritic cells, keratinocytes, and endothelial cells [94]. As the sole ligand for the receptor CXCR6, soluble CXCL16 promotes the directional migration of CXCR6^+^ cells, such as CD4^+^ effector memory T cells and natural killer T-cells [95], with expression being induced by inflammatory cytokines [96]. All sites under selection in our study are in the extracellular section of the transmembrane polypeptide, indicating possible functional changes in the interactions between CXCL16 and its receptor, CXCR6.

From among the genes identified in the selective sweep analysis, *ANKRD17* had the highest number of codons under selection. This gene encodes for an ankyrin repeat protein, which has previously been reported to be an important regulator of the cell cycle [97] and may also be involved in innate immune activation by viruses and bacteria [98, 99]. The codons under selection for this gene are clustered in the C-terminus of the protein, outside of the region of ankyrin repeats that has been shown to bind to the NLR-family pattern-recognition receptor, NOD2 [99], and in the region that is putatively responsible for activation of RIG-I-like receptors [98]. This may indicate some cross-talk between antifungal and antiviral innate signalling pathways, or it could be related to the presence of viral coinfections in some bats with WND [100]. Our literature search identified several studies that implicated the involvement of ANXA1, Annexin A1, a protein that is a well-characterized anti-inflammatory inhibitor of cytosolic phospholipase A2, in a variety of human diseases [101]. In addition, *ANXA1* is a known target for positive selection in *Myotis* infected by *P. destructans* [33], and shows increased transcription in hibernating, infected Nearctic *M. lucifugus* compared to uninfected conspecifics, or Palearctic *M. myotis* [27]. Harazim et al. [33]. suggested *ANXA1* may act via two different routes in bats infected by *P. destructans*. First, ANXA1 has the potential to down-regulate the immune responses initiated by bats arousing from torpor, which can lead to immunopathology if left unchecked. ANXA1 appears to regulate the neutrophil response under fungal infection conditions, altering lipid membrane organization and metabolism [102]. ANXA1 has also been found to participate in adaptive immunity against chronic infectious disease [103], by directing the immune response towards a Th1/Th17 response, a response that is associated not only with clearing fungal infection in normothermic mammals, but also the mortality evidenced in susceptible Nearctic populations of *M. lucifugus* infected by *P. destructans* [17]. Second, ANXA1 also appears to play a role in wound repair and epithelial recovery, extending its importance beyond the acute phase of inflammation to the equally important healing phase [104, 105]. Our study found five of the seven sites recognized under positive selection in the study by Harazim et al. [33]. , with an addition of four previously undescribed sites. All previously identified sites were confined to the N-terminal region of the protein, where ANXA1 is known to bind to S100A11 and inhibit phospholipase A2 [106, 107]. We found additional evidence for selection at three sites towards the C-terminal end of the protein, whose function is not as well characterized.

We acknowledge that our screen for selection filters out other types of adaptive variation that may play a key role in the development of tolerance. The screen we employ here fails to take into account the role synonymous codon changes have in altering mRNA structure and stability, the rate at which translation occurs and any resulting structural changes in protein conformation [108, 109]. Additionally, by primarily screening protein coding genes any adaptive variation in introns, UTRs and intergenic regions that may potentially alter the expression [110] of key proteins are excluded from our analysis.

Our combined approach to identify genes involved in adaptation to infection by *P. destructans* revealed a preponderance of genes with known functions involved in defence responses, including both innate and adaptive immune responses. In addition to the specific genes described above, we found strong evidence that pathways that activate inflammatory responses and neutrophil recruitment are under selection. This is highly consistent with the observed pathology associated with infection by *P. destructans* in naive populations, both during hibernation and after emergence [111]. IL-8 signalling appears to be among the inflammatory pathways that are particularly important, and this cytokine is known to recruit neutrophils to sites of infection and enhance their activation, a process that likely generates significant immunopathology. Other pathways that appear to be involved in adaptation included coagulation and stress responses, although it is not clear how increased or decreased activation of these pathways would enhance disease tolerance. Presumably the driving force of natural selection is to suppress the inflammatory responses that lead to immunopathology, or at least delay them until it is more energetically favourable to mount a resistance response.

## Conclusions

A volume of research over the last decade indicates not only the significance of immunopathology in the cascade of physiological events that build up to mortality associated with WND [15–17], but also the lack of immunopathology in infected hosts in the Palearctic [25–27, 36]. This is suggestive of the evolution of tolerance in Palearctic bats through coevolution over an extended period of time [23, 29], a process that may already have been initiated in Nearctic *Myotis* also [112, 113]. Selection towards tolerance to the fungal infection in the Palearctic has been suggested [5, 29] and indicated at a genomic level [33], but here, through an exhaustive approach, we can state with higher confidence that genes and pathways associated with fungal infections, and particularly those involved in infection by *P. destructans*, have been under positive selection in Palearctic species of *Myotis*. Of course, when investigating patterns of selection across entire genomes of bats, albeit related, across two continents, we acknowledge that there are a plethora environmental factors also driving the observed selection patterns. Therefore, further functional analysis e.g., proteomics and immunological modelling, or experimental validation is needed to elucidate exactly how the observed selection affects response to infection. Nevertheless, attempts to identify these genes, and their variants will allow for the estimation of survival in North American affected species through population genetic approaches to assist in implementing conservation measures [5, 114]. Finally, not only do the results illuminate processes driving tolerance in a bat fungal disease, but bolster our holistic understanding of how novel host-parasite interactions drive selection towards tolerance in host organisms.

### Electronic supplementary material

Below is the link to the electronic supplementary material.

Supplementary Material 1

Supplementary Material 2

Supplementary Material 3

Supplementary Material 4

Supplementary Material 5

Supplementary Material 6

Supplementary Material 7

Supplementary Material 8

Supplementary Material 9

Supplementary Material 10

Supplementary Material 11

Supplementary Material 12

Supplementary Material 13

Supplementary Material 14

Supplementary Material 15

Supplementary Material 16

Supplementary Material 17

Supplementary Material 18

Supplementary Material 19

Supplementary Material 20

Supplementary Material 21

Supplementary Material 22

Supplementary Material 23

Supplementary Material 24

Supplementary Material 25

Supplementary Material 26

Supplementary Material 27

Supplementary Material 28

Supplementary Material 29

Supplementary Material 30

Supplementary Material 31

Supplementary Material 32

Supplementary Material 33

## Data Availability

All raw data generated in this study are accessible at NCBI under bioproject PRJNA1051501. All final gene alignments, and gene trees can be found on Zenodo at 10.5281/zenodo.11353518.
