# Supplementary material for: Signals of positive selection in genomes of palearctic Myotis-bats coexisting with a fungal pathogen
Source: BMC Genomics. 2024 Sep 3;25:828. doi: 10.1186/s12864-024-10722-3 (PMC11370307; doi:10.1186/s12864-024-10722-3)
Supplement: Supplementary file 2 — Supplementary Material 2 [file 12864_2024_10722_MOESM2_ESM.docx]

Supplementary Figure 1: Enrichment analysis for the phylogenetic dataset

Supplementary Figure 2: Underrepresentation analysis for the phylogenetic dataset

Supplementary Figure 3: Proportion of genes for each omega value for the A) Phylogenetic dataset and b) Curated gene dataset, blue line represents the median value

Supplementary Figure 4: Selection signatures identified by μ statistics from RAiSD with selection significance cutoff (0.05%, red line) in *Myotis lucifugus*

Supplementary Figure 5: Selection signatures identified by μ statistics from RAiSD with selection significance cutoff (0.05%, red line) in *Myotis myotis*.

Supplementary Figure 6: A) Summary of nucleotide diversity, Watterson’s Theta, and Tajima's D in *Myotis lucifugus* and *Myotis myotis*; B) Wattersons’ Theta and Tajima’s D by scaffold in *Myotis lucifugus* and C) in *Myotis myotis*

Supplementary Figure 7: Location of the TNFSF4 transmembrane domain and location of positively selected sites

Supplementary Figure 8: Relative solvent availability plot of the modelled section of TNFSF4

Supplementary Figure 9: Location of the CXCL16 transmembrane domain and positively selected sites

Supplementary Figure 10: Similarity plot showing the variation between species along the ANKRD17 gene alignment. Higher values indicate higher conservation, sliding window size was 20 nt

Supplementary Figure 11: Relative solvent availability plot of ANXA1

Supplementary Figure 12: Phylogenetic tree showing the ASTRAL quartet scores

Supplementary Table 1: Sample collection details

Supplementary Table 2: Details of data downloaded from NCBI

Supplementary Table 3: Gene information for the 2,515 genes of the phylogenetic dataset

Supplementary Table 4: Gene information for A) genes identified in the literature search, B) Genes linked to selective sweep analysis and C) Genes in the top 1% Omega values under the null model (branch test)

Supplementary Table 5: Gene information for the 300 genes in the curated gene dataset

Supplementary Table 6: Summary of sequencing and assembly statistics for all samples

Supplementary Table 7: Summary tables of paml branch tests for the phylogenetic dataset

Supplementary Table 8: Summary table of SNP calling done with ANGSD and GATK and the overlapping SNPs from both of the callers for *Myotis lucifugus* and *Myotis myotis* with observed and expected homozygosity and the inbreeding coefficient

Supplementary Table 9: Selective sweep positions above significance thresholds (0.05%) with corresponding gene annotations for *Myotis lucifugus*

Supplementary Table 10: Selective sweep positions above significance thresholds (0.05%) with corresponding gene annotations for *Myotis myotis*

Supplementary Table 11: Summary tables of paml codeml tests for the curated gene dataset

Supplementary Table 12: Overall summary of the curated gene dataset A) Omega values and B) significant in all nested model comparisons (codon test)

Supplementary Table 13: Summary of all significant amino acid sites under selection for the 38 genes identified in all codon tests as significant

Supplementary Table 14: gprofiler results for the 38 significant genes from the curated gene dataset

Supplementary Methods 1: SDS DNA extraction method details

Supplementary Model 1: A pdb file containing the model for TNFSF4

Supplementary Model 2: A pdb file containing the model for ANXA1

Supplementary File 1: Movie showing a 360^o^ view of the modelled structure of TNFSF4, sites under selection are coloured

Supplementary File 2: Movie showing a 360^0^ view of the modelled ANXA1 structure, with binding sites and sites under selection as highlighted in figure 5.
