# Supplementary figures and images for: Signals of positive selection in genomes of palearctic Myotis-bats coexisting with a fungal pathogen
Source: BMC Genomics. 2024 Sep 3;25:828. doi: 10.1186/s12864-024-10722-3 (PMC11370307; doi:10.1186/s12864-024-10722-3)

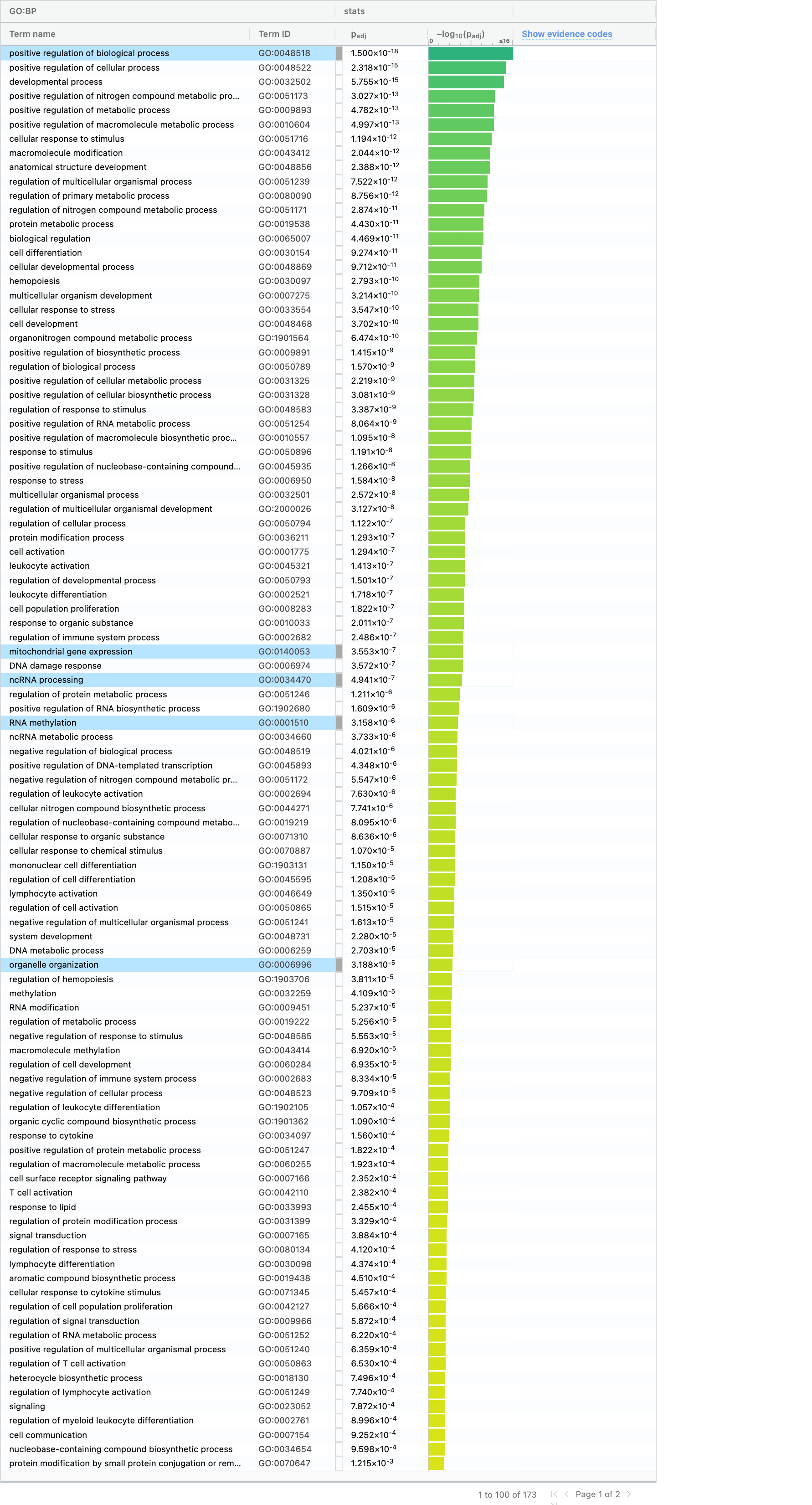

Supplement: Supplementary file 3 — Supplementary Material 3 [file 12864_2024_10722_MOESM3_ESM.png]

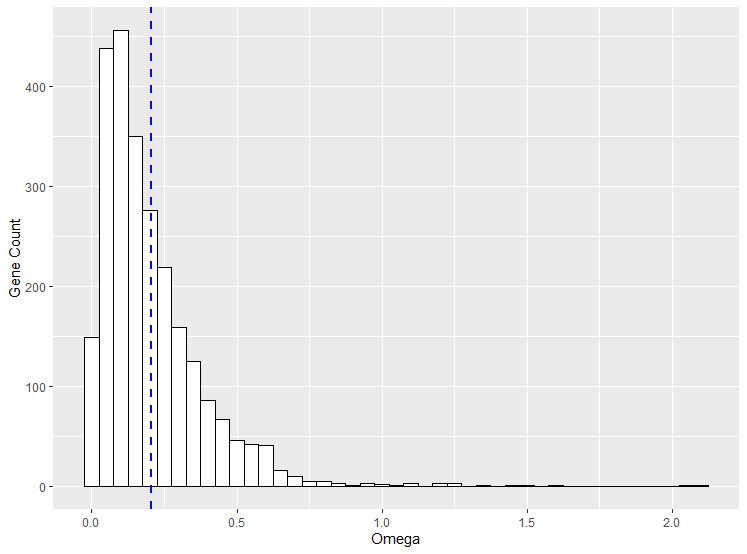

Supplement: Supplementary file 4 — Supplementary Material 4 [file 12864_2024_10722_MOESM4_ESM.png]

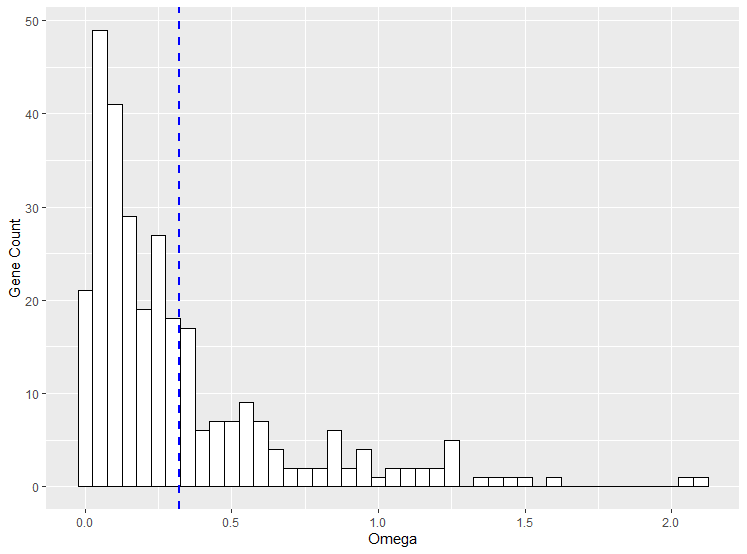

Supplement: Supplementary file 5 — Supplementary Material 5 [file 12864_2024_10722_MOESM5_ESM.png]

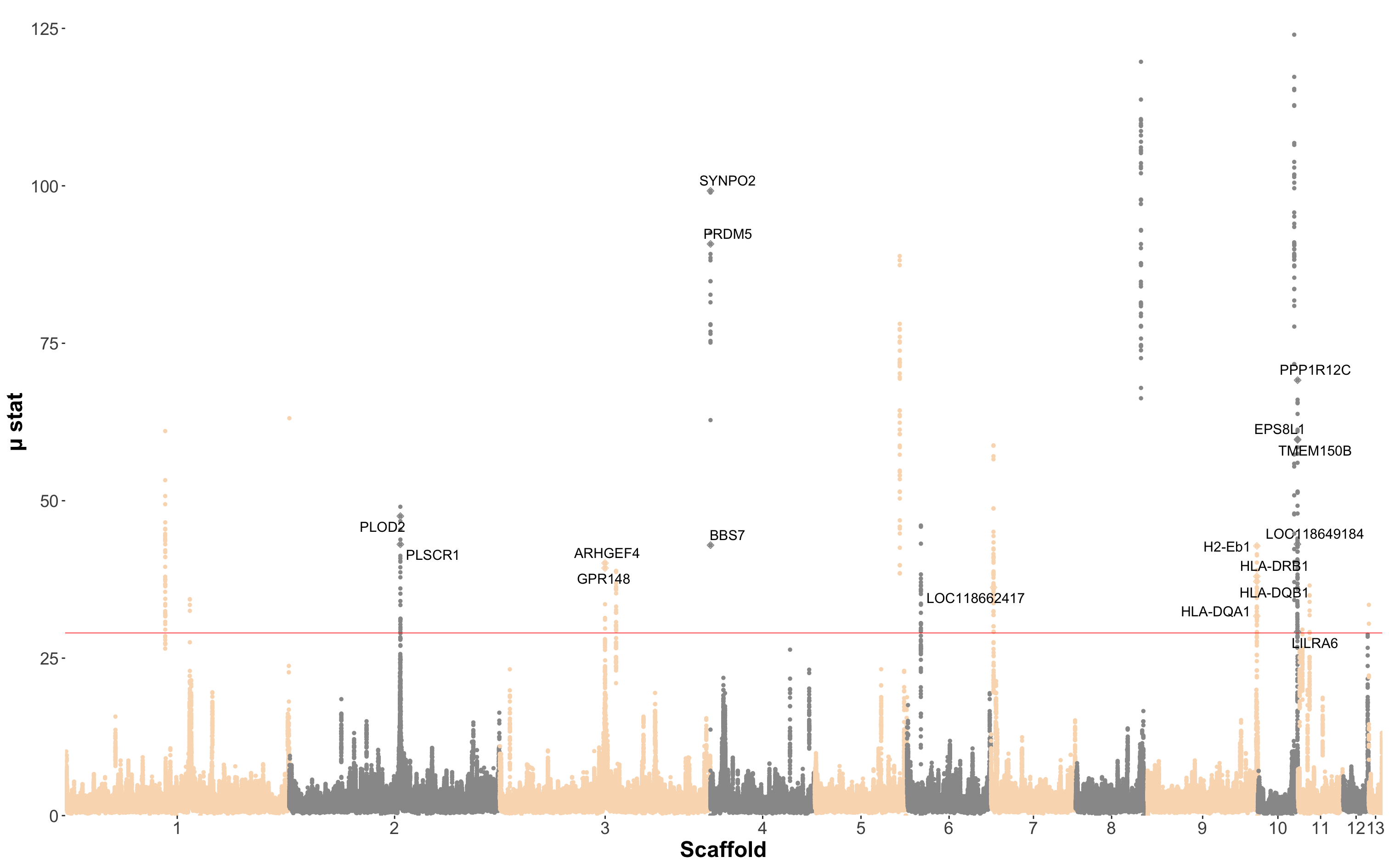

Supplement: Supplementary file 6 — Supplementary Material 6 [file 12864_2024_10722_MOESM6_ESM.png]

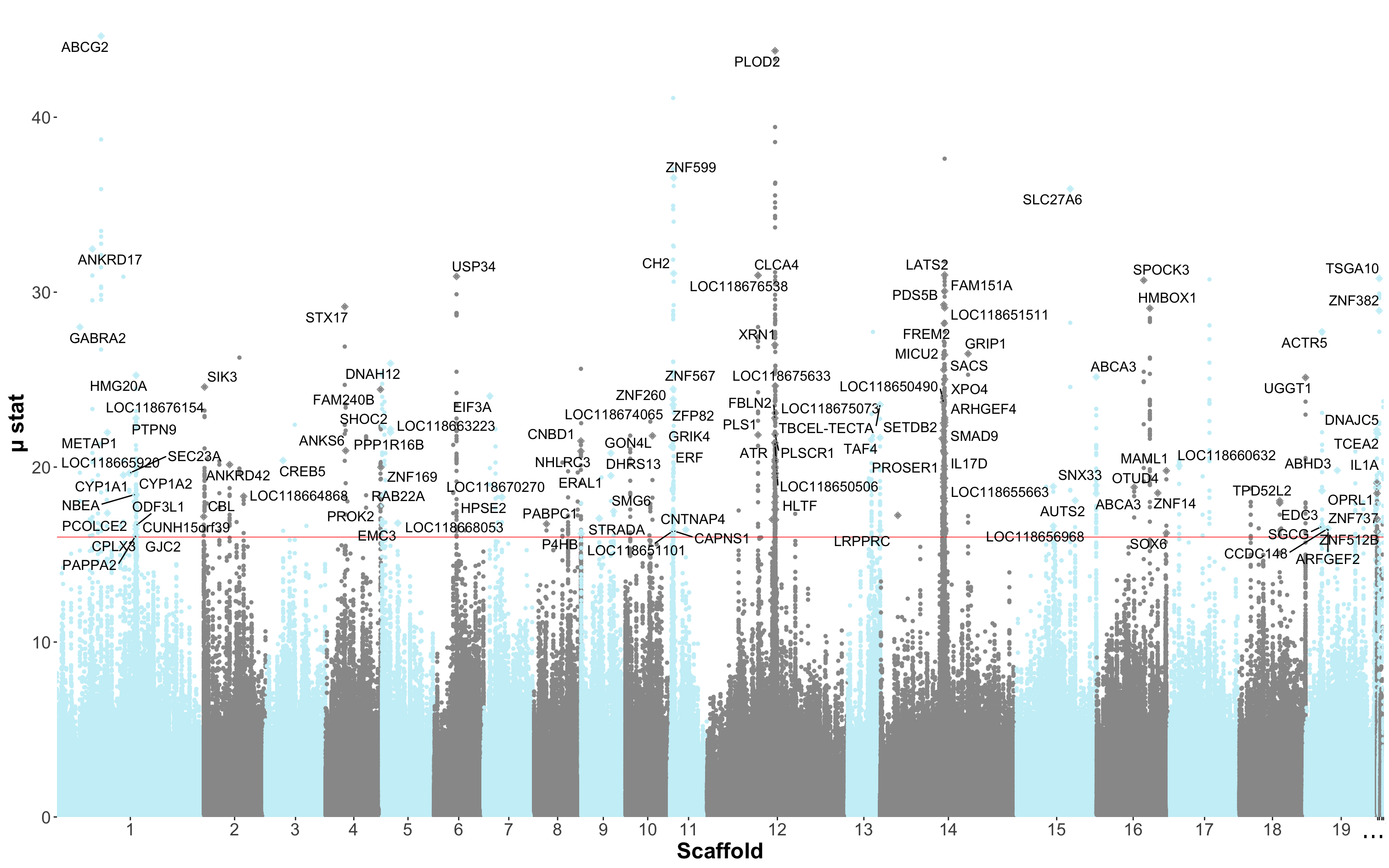

Supplement: Supplementary file 7 — Supplementary Material 7 [file 12864_2024_10722_MOESM7_ESM.png]

A

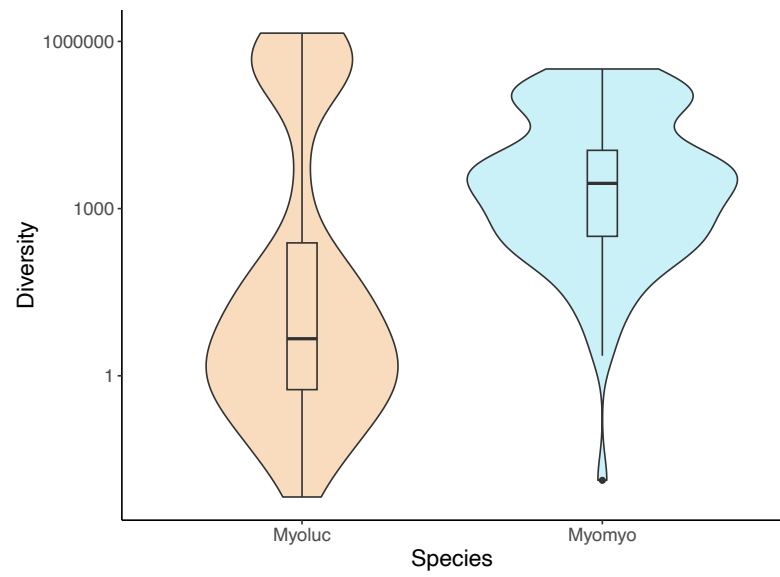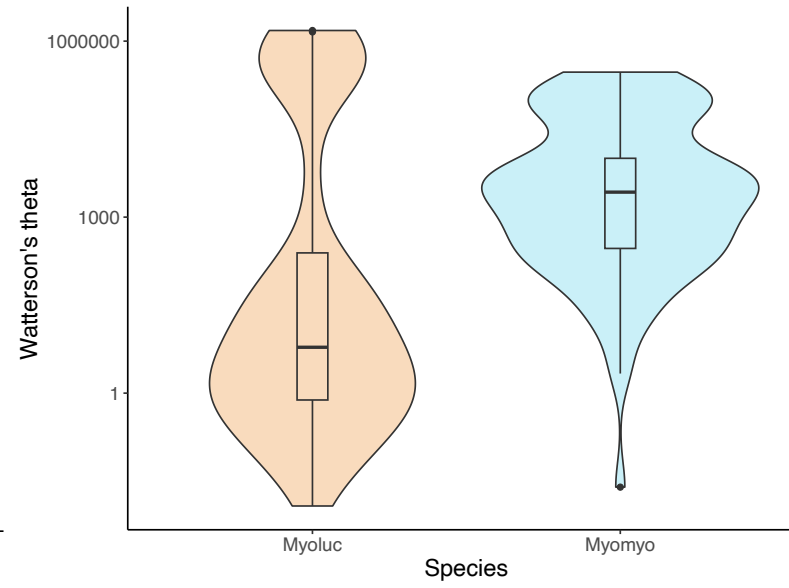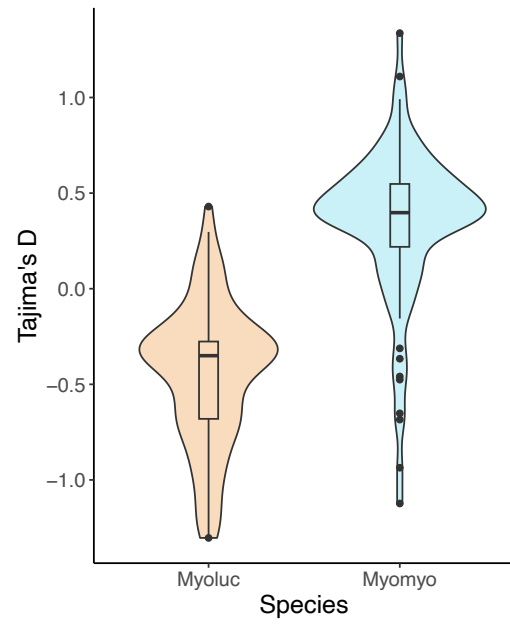

B

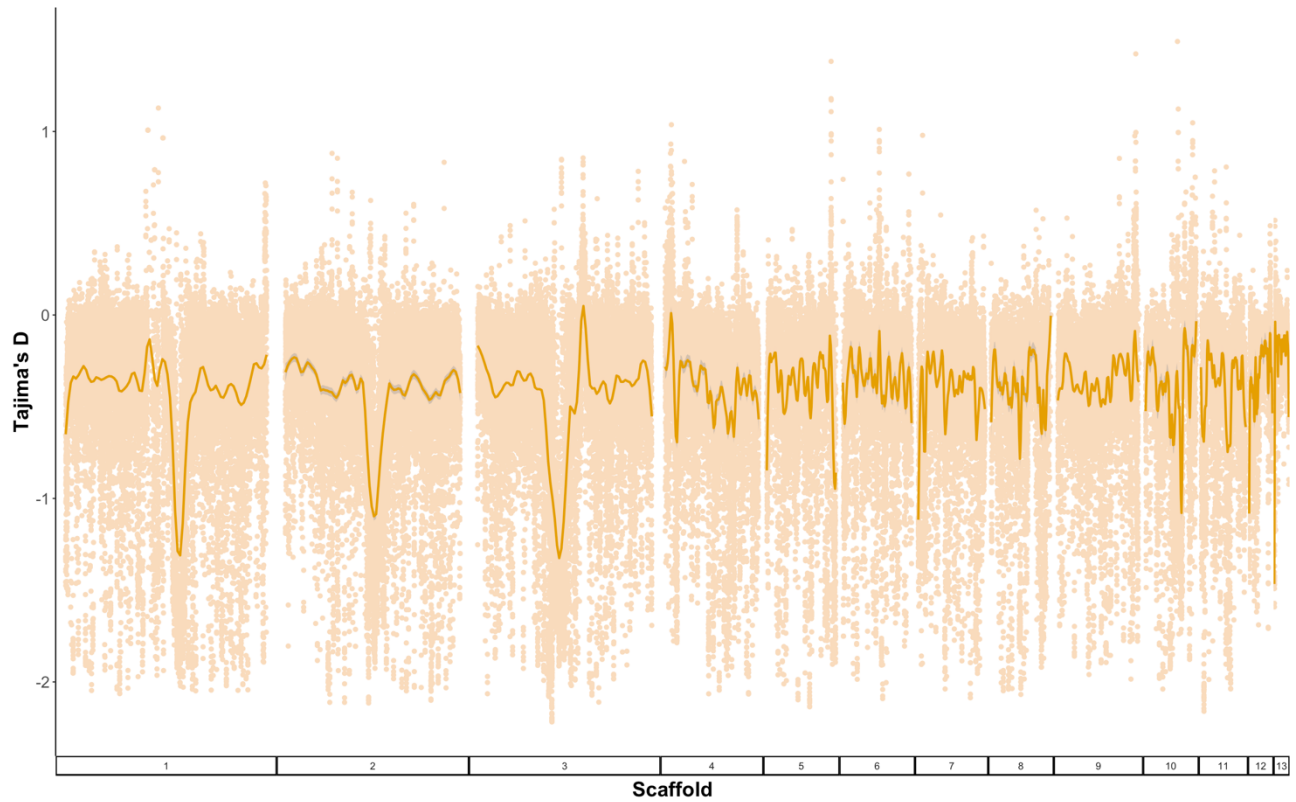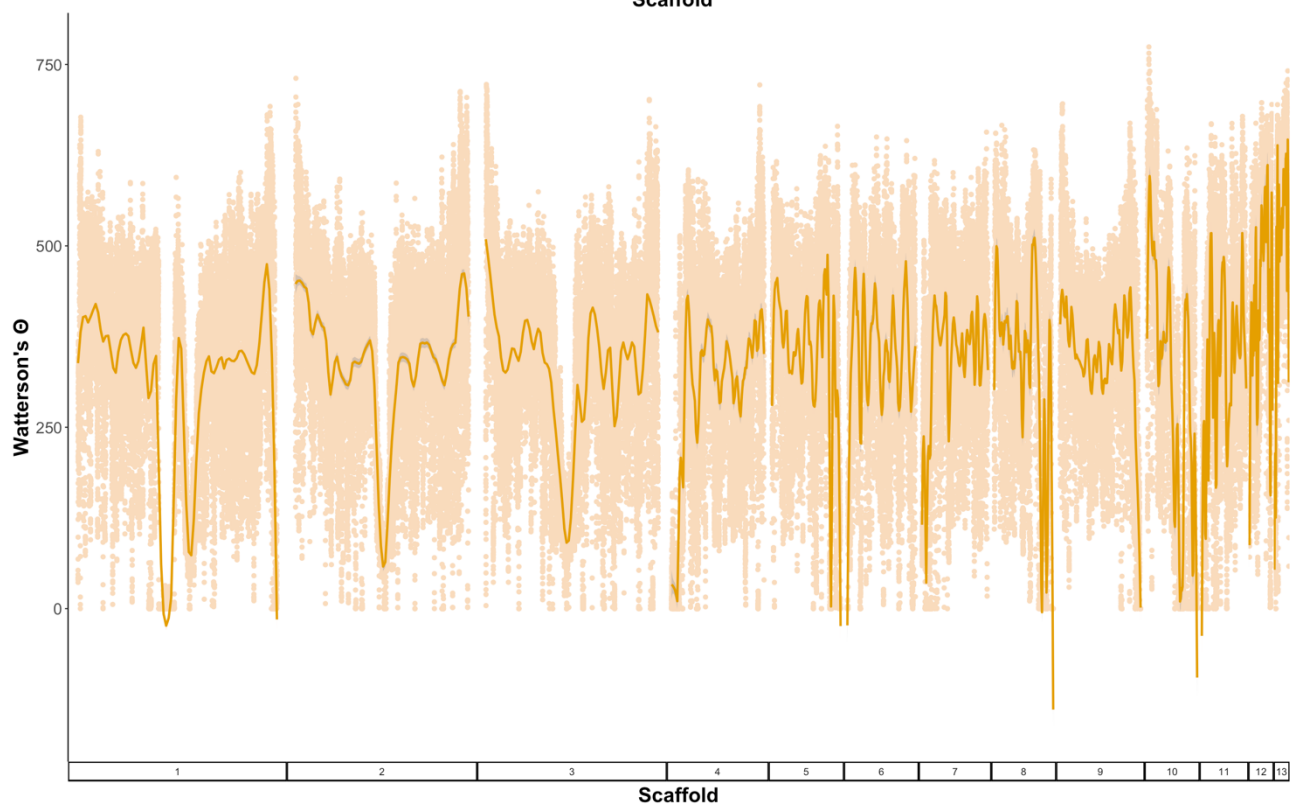

C

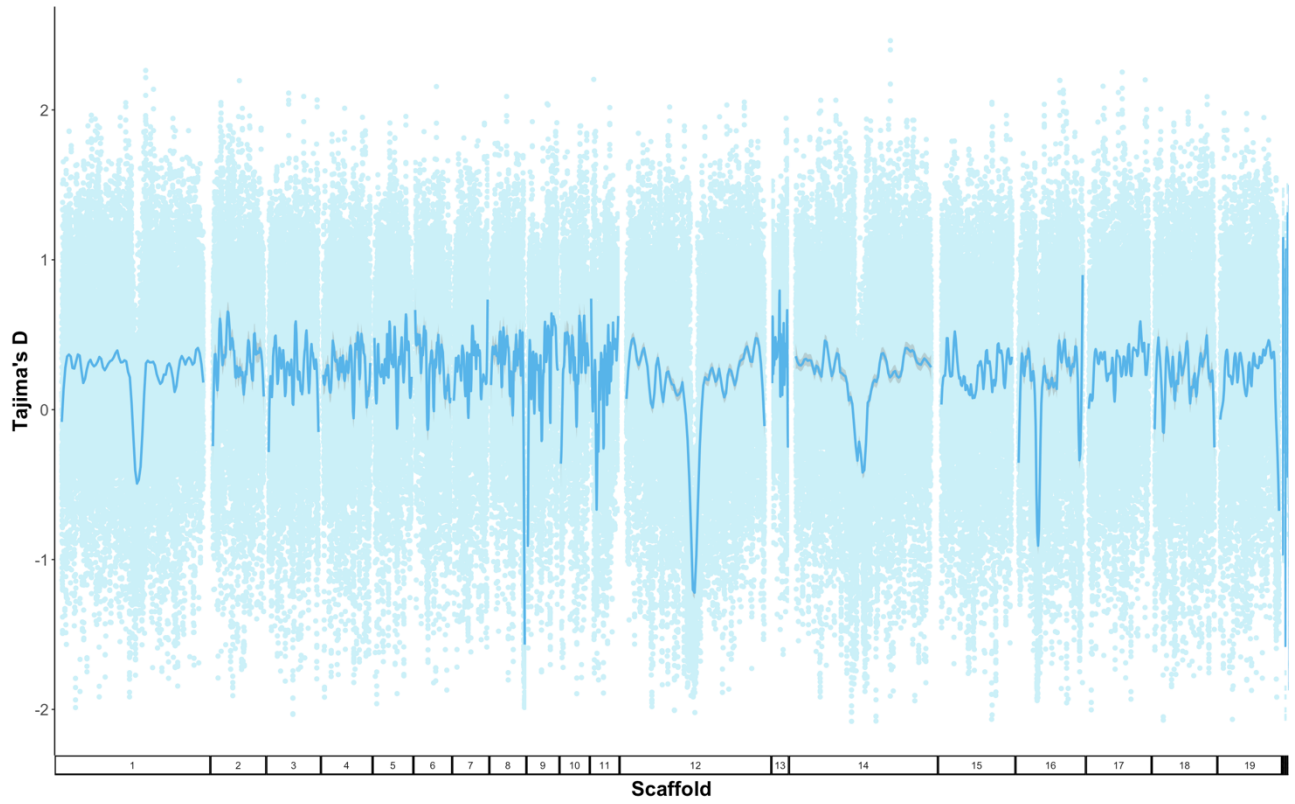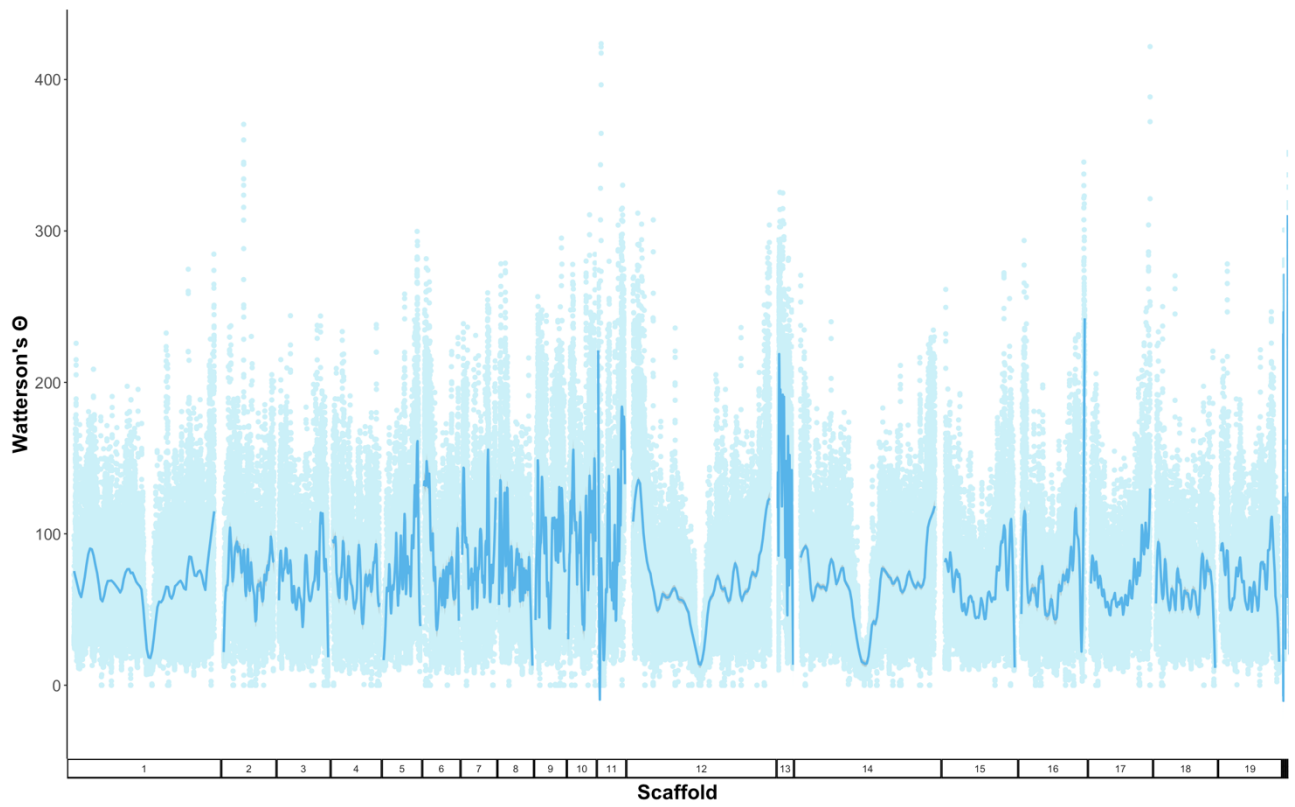

Supplement: Supplementary file 8 — Supplementary Material 8 [file 12864_2024_10722_MOESM8_ESM.pdf]

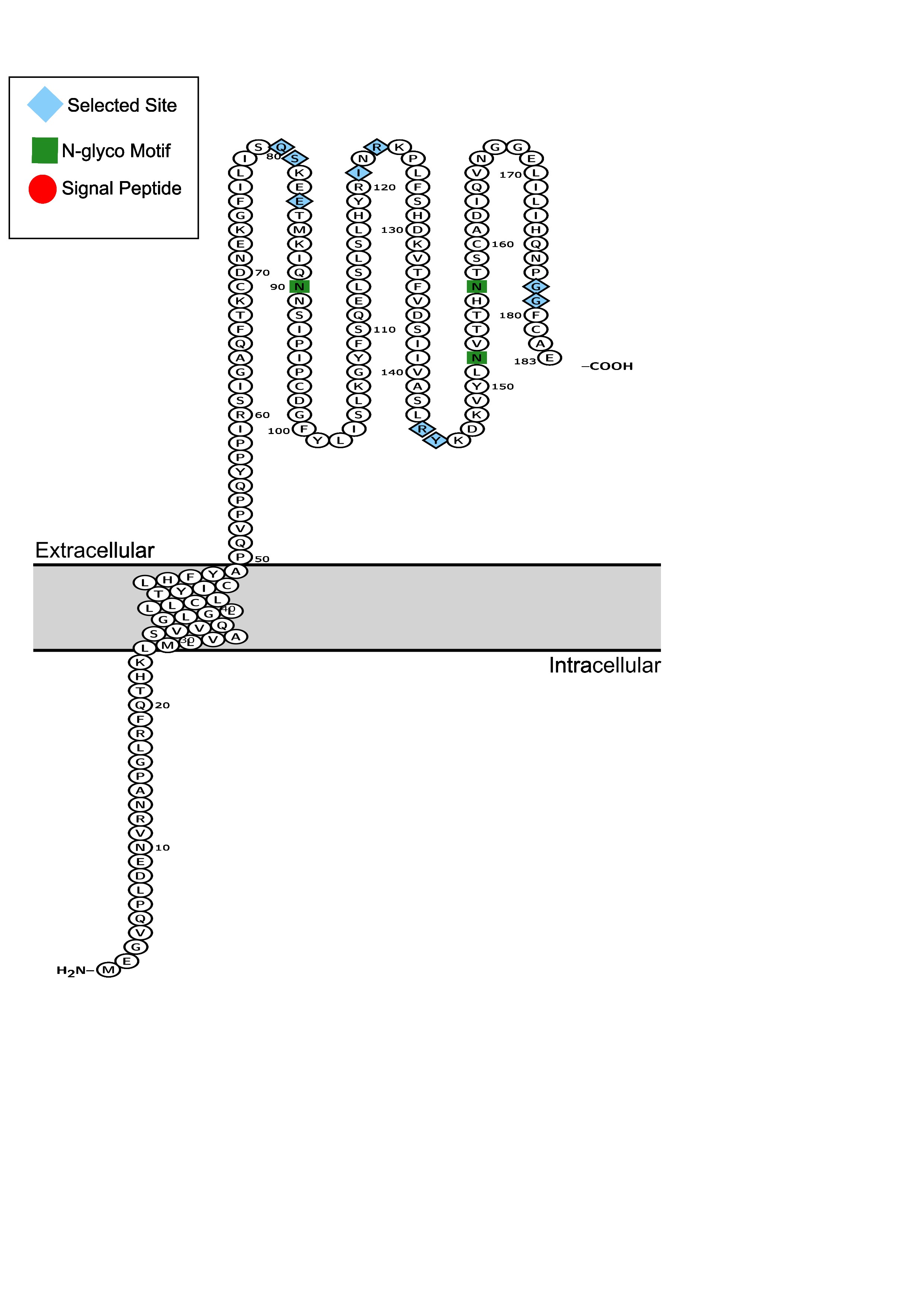

Supplement: Supplementary file 9 — Supplementary Material 9 [file 12864_2024_10722_MOESM9_ESM.jpg]

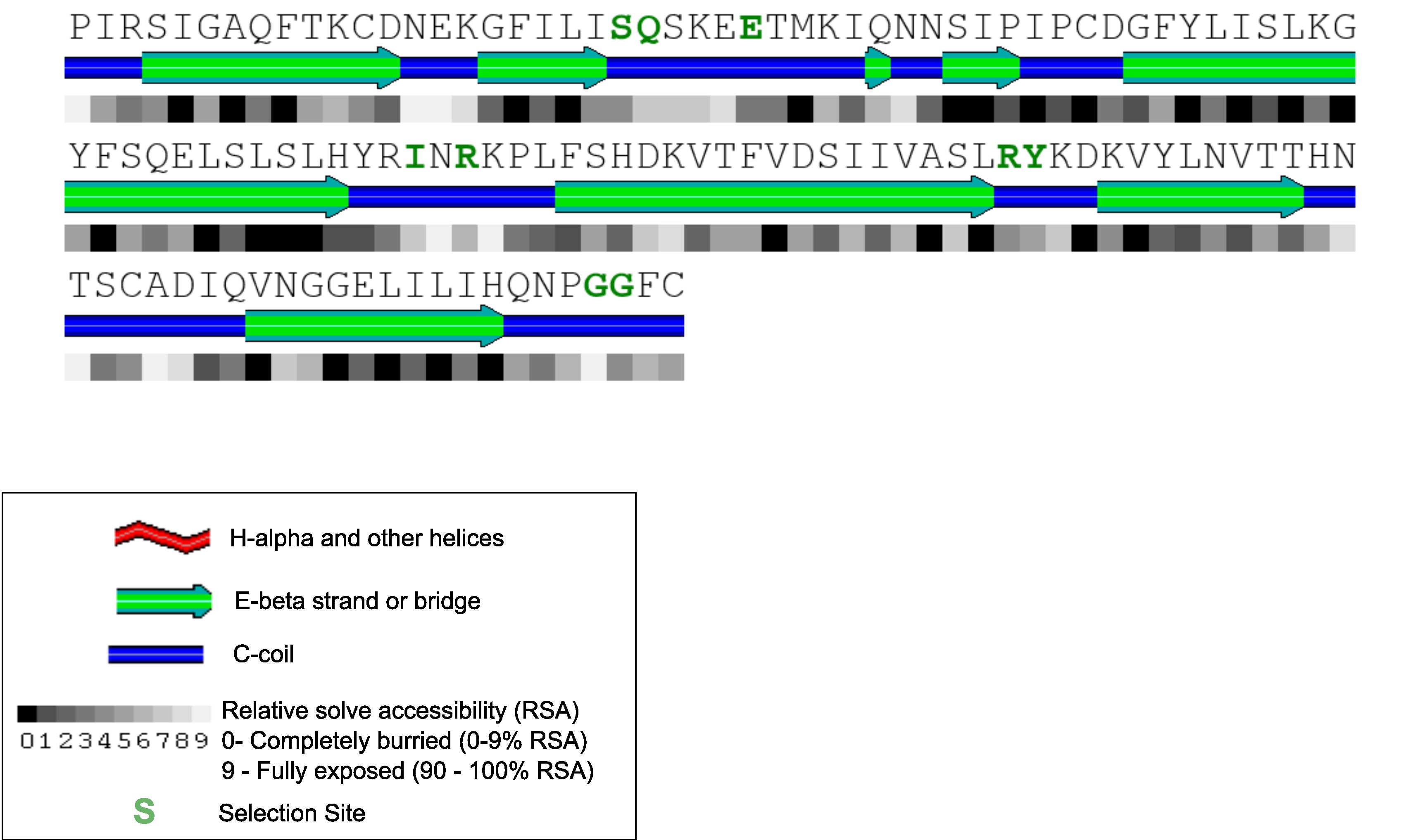

Supplement: Supplementary file 10 — Supplementary Material 10 [file 12864_2024_10722_MOESM10_ESM.jpg]

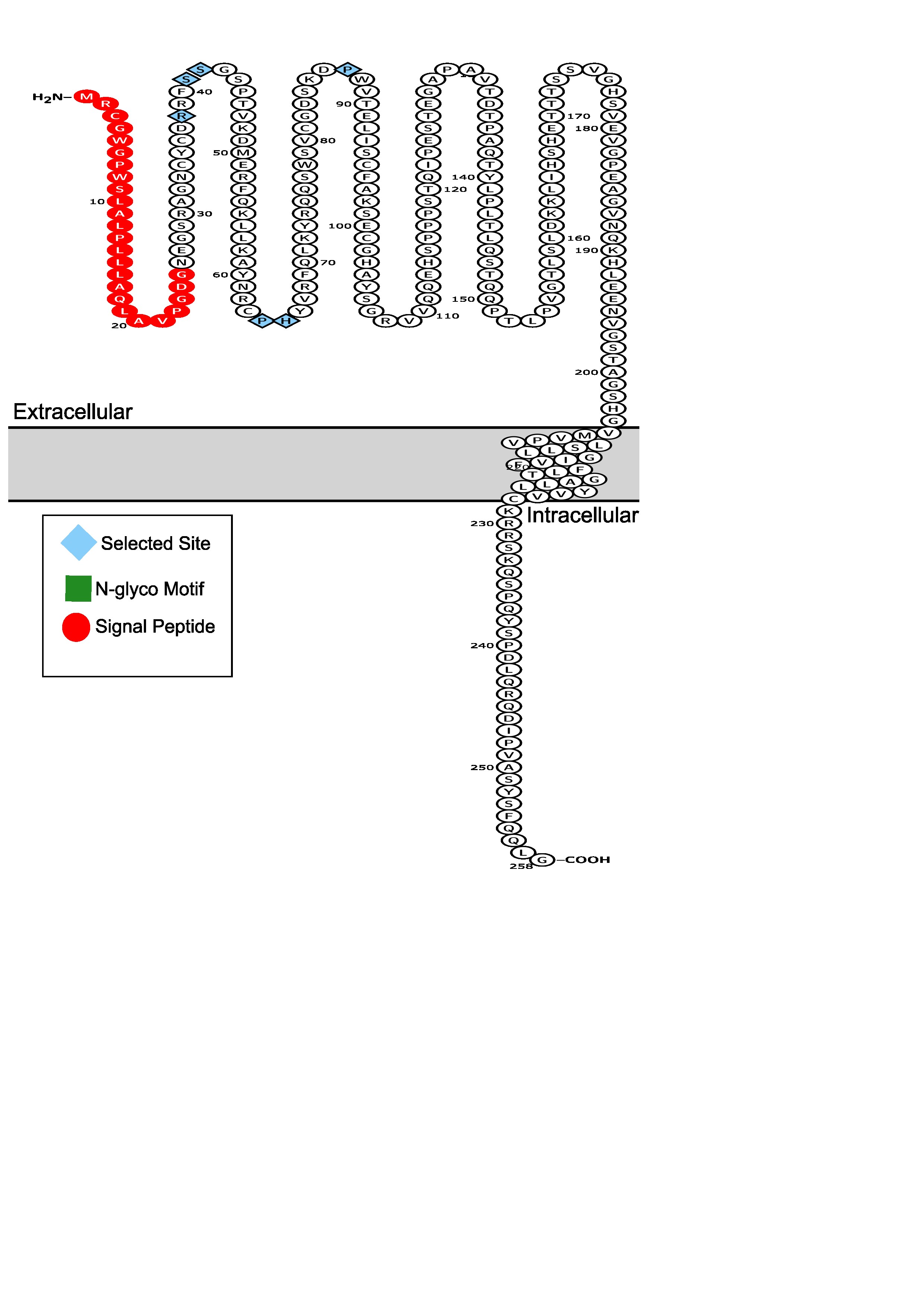

Supplement: Supplementary file 11 — Supplementary Material 11 [file 12864_2024_10722_MOESM11_ESM.jpg]

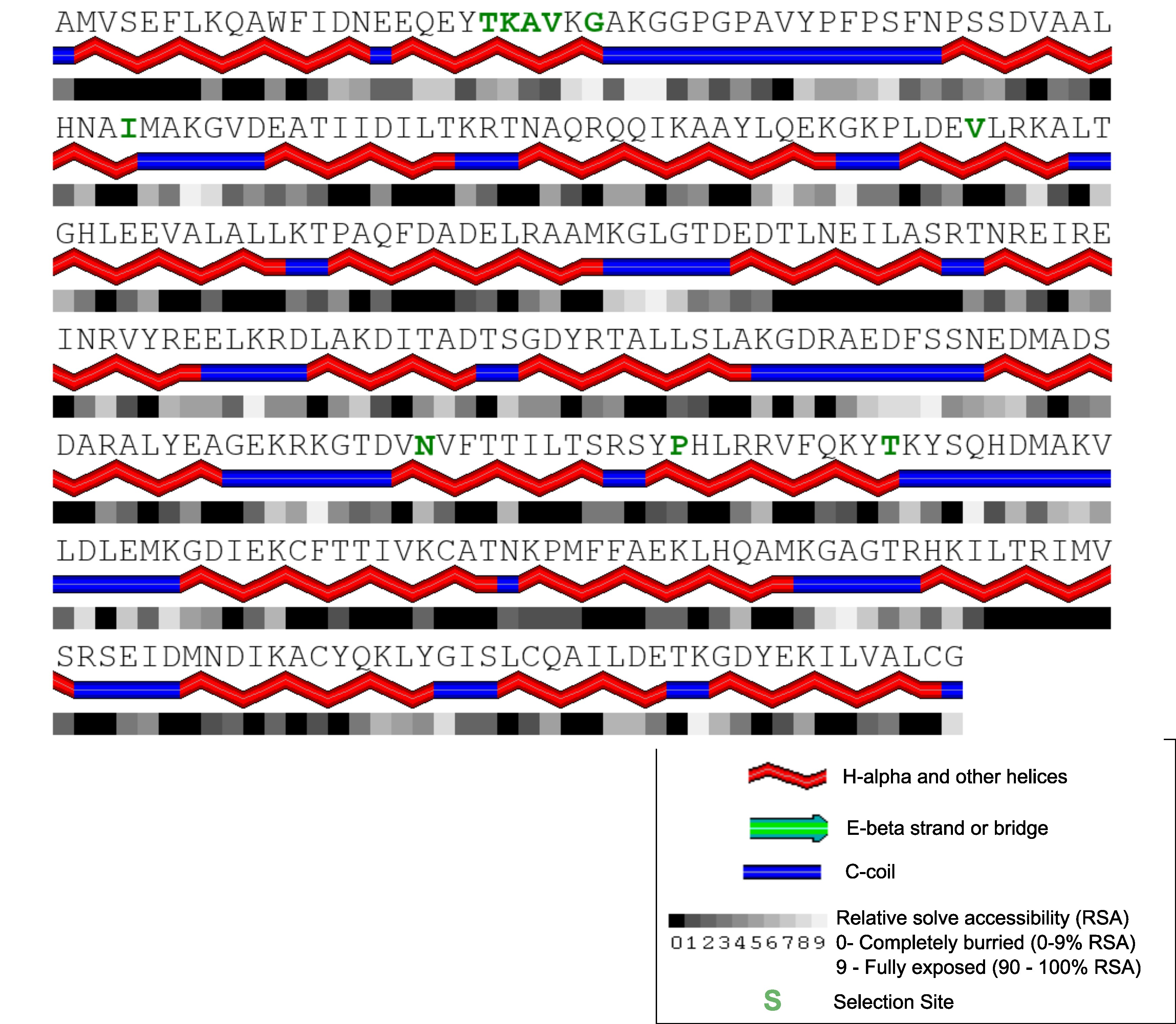

Supplement: Supplementary file 13 — Supplementary Material 13 [file 12864_2024_10722_MOESM13_ESM.jpg]

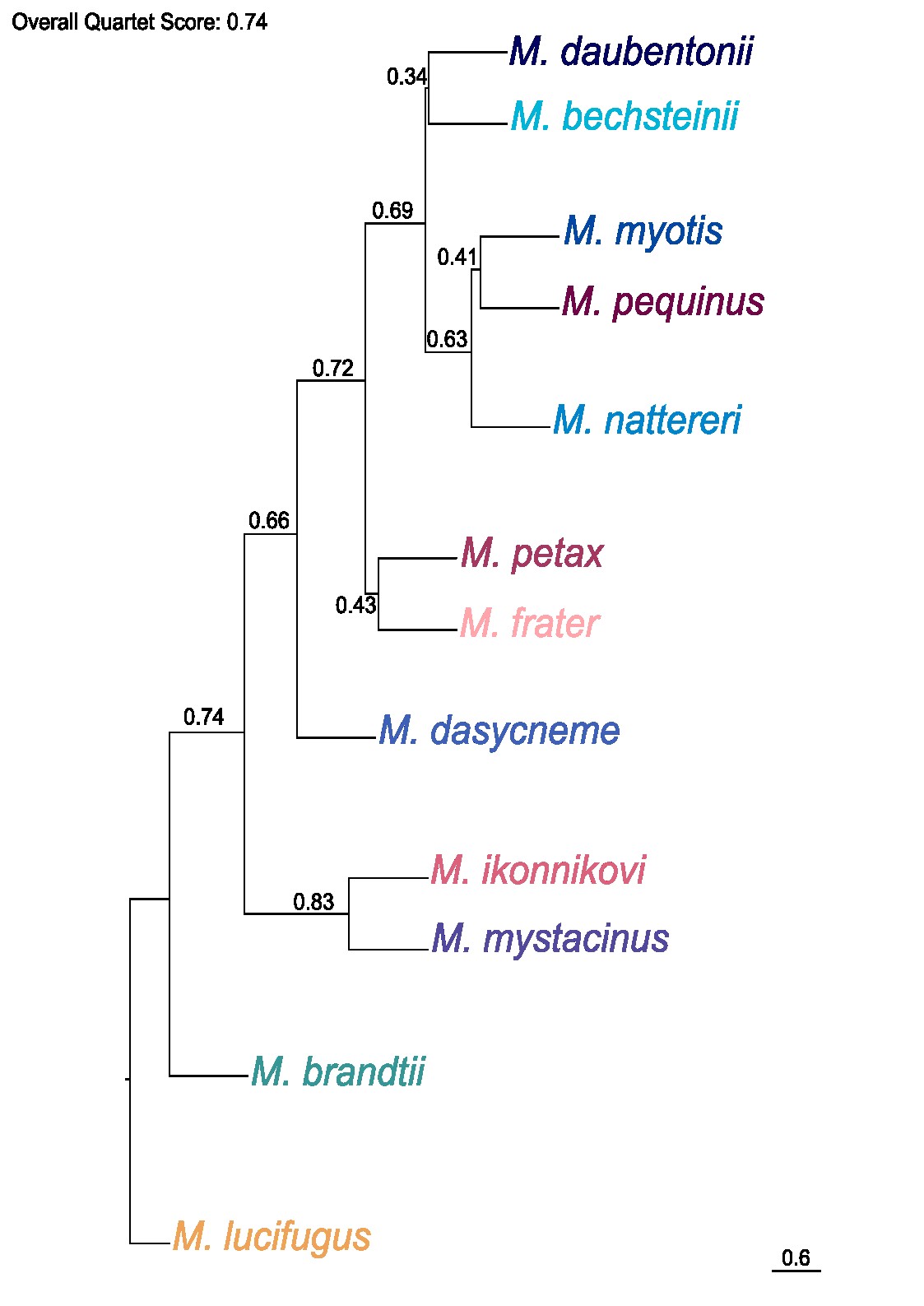

Supplement: Supplementary file 14 — Supplementary Material 14 [file 12864_2024_10722_MOESM14_ESM.jpg]
