# Supplementary material for: Signals of positive selection in genomes of palearctic Myotis-bats coexisting with a fungal pathogen
Source: BMC Genomics. 2024 Sep 3;25:828. doi: 10.1186/s12864-024-10722-3 (PMC11370307; doi:10.1186/s12864-024-10722-3)
Supplement: Supplementary file 17 — Supplementary Material 17 [file 12864_2024_10722_MOESM17_ESM.pdf]

SDS Extraction Methods

I. Purpose

Extraction of sample DNA.

II. Scope

This standard operating procedure (SOP) is applicable to the extraction of DNA from animal samples. The DNA extraction process of non-such samples is different from this SOP, and must be carried out in accordance with the SOP for DNA extraction of other samples.

III. Principle

SDS is an anionic surfactant that solubilizes membrane proteins to disrupt cell membranes and denature the proteins to precipitate them.

IV. Experimental instruments

Product Name	Manufacturer	Model number
High-speed centrifuge	Thermo	Fresco 17
Water bath	Shanghai Jinghong Experimental Equipment Co., Ltd.	DK-8D
Oscillator	DHS Life Science & Technology Co., Ltd.	VS-1
-20°C Refrigerator	Shanghai Jingxin Industrial Development Co., Ltd.	BCD-301WT/A
Multi-sample tissue grinder	Shanghai Jingxin Industrial Development Co., Ltd.	-
Pipette	eppendorf	0.5-10 μl, 20-200 μl, 100-1000 μl
Pipette	eppendorf	20-200 μl
Pipette	eppendorf	100-1000 μl

V. Experimental reagents and consumables

Name	Brand name	Specifications	Item No.
concentrated hydrochloric acid (HCl)	Beijing Chemical Factory Co., Ltd.	500 ml	-
NaOH	Made in China	500 g	cc-qyhn
SDS	Made in China	500 g	-
Nacl	Made in China	500 g	-
Tris saturated phenol	Solarbio	250 ml	-
Chloroform	Beijing Chemical Factory Co., Ltd.	500 ml	-
Isopentyl alcohol	Tianjin Damao Chemical Reagent Factory	500 ml	-
Isopropyl alcohol	Made in China	500 ml	-
Anhydrous ethanol	Tianjin Damao Chemical Reagent Factory	500 ml	-
Tris	amresco	500 g	-
EDTA	Beijing Chemical Factory Co., Ltd.	250 g	-
Proteinase K	Roche	100 mg	3508838103
lysozyme	Tiangen Biotech (Beijing) Co., Ltd.	1 ml	RT401
RNase A	Tiangen Biotech (Beijing) Co., Ltd.	1 ml	RT405
EB	QIAGEN	250 ml	19086
Ball bearing	Made in China	-	-

VI. Operating considerations

1. The whole operation must wear lab coats, masks, PE gloves, and latex gloves.
2. Before operation, distinguish between the contaminated area and the non-contaminated area, and change gloves after operation in the contaminated area to prevent the non-contaminated area from being contaminated.
3. Sampling on dry ice.
4. Tris water-saturated phenol is easily oxidized by air and turns pink; such phenol is easy to degrade DNA and generally cannot be used. Normally stored in the refrigerator at 4 °C, when used, open the lid to absorb the cover quickly so that the phenol does not deteriorate and is available for several months.
5. When preparing 1% SDS, 10% SDS is added last to avoid a large number of bubbles. Pay attention to the bubbles during the finalization and wait for the bubbles to subside before finalizing.
6. Check the sample name and number after sampling to ensure that the sample information is correct.

VII. Experimental procedures

1. Sampling:

a. Prepare grinding tubes (1 steel ball per tube) according to the work order, place the sample on PE gloves and cut about 50 mg in the grinding tube, and quickly put it into liquid nitrogen to avoid degradation (sampling on dry ice; change one set of PE gloves and blades for each sample).

b. Pre-cool the grinder module, put the sample tube together with the module into the grinder, and adjust the parameters (general frequency 45-50 times/30s) for grinding.

c. Confirm that the samples grinding is sufficient to transfer the samples on the plate, and then add 550 ul 1% SDS lysate, 150 ul 0.5M EDTA, 30 ul Proteinase K, and 2% mercaptoethanol, respectively, invert to mix well, and incubate at 55°C for 30-180 min (different tissues lysis times are different: the cell 180 min; flounder, liver, etc., 40 min, ear 90 min).

2. Centrifuge at 12500 rpm for 5 min, aspirate the supernatant into a new 1.5 ml EP tube, add 300 ul of protein precipitation solution (5M NaCl), leave it at 20 °C for 5 min, and centrifuge at 12500 rpm for 5 min.
3. Aspirate the supernatant into a new 1.5 ml EP tube, and centrifuge at 12500 rpm for 5 min, there may still be a trace of protein precipitate at the bottom of the tube.

4. Aspirate the supernatant into a new 2 ml EP tube, add an equal volume of saturated phenol, chloroform, and isoamyl alcohol (25: 24: 1), mix upside down for 5 min, and centrifuge at 12500 rpm for 7 min.

5. Carefully aspirate the supernatant into a new tube (preferably sparingly, the tip of the gun should not touch the stratified area), add an equal volume of chloroform and isoamyl alcohol, mix gently and then centrifuge at 12,500 rpm for 8 min.

6. Aspirate the supernatant into a new 1.5 ml EP tube, add 3/4 volume of isopropanol, mix well and leave at -20 ℃ for 20 min (-80 ℃ for 10 min), centrifuge at 12000 rpm for 10 min.

7. Pour out the liquid, taking care not to pour out the precipitate. Wash twice with 1 ml of 75% ethanol, the remaining small amount of liquid can be collected by centrifugation again, and then sucked out with a gun tip.

8. Blow-dry on an ultra-clean bench or air-dry at room temperature (DNA samples should not be too dry, or they will be difficult to solubilize).

9. Add 100 ul EB to dissolve the DNA sample.

10. Add 2 ul RNase A, mix upside down, and incubate at 37 ℃ for 15 min.
